# Fracture patterns in diaphyseal gunshot trauma: role of the bullet’s impact point and angle

**DOI:** 10.1007/s00414-025-03488-0

**Published:** 2025-04-07

**Authors:** Nathalie Schwab, Doreen Jost, Xavier Jordana, Jordi Monreal, Xavier Garrido, Pedro Brillas, Ignasi Galtés

**Affiliations:** 1 Institute of Legal Medicine St.Gallen, Cantonal Hospital, HOCH Health Ostschweiz, University teaching and research hospital, St.Gallen, 9007 Switzerland; 2 Forensic Anthropology Unit, Forensic Pathology Service, Catalonian Institute of Legal Medicine and Forensic Science (IMLCFC), Ciutat de la Justícia, Gran Via de les Corts Catalanes, 111 Edifci G, Barcelona, 08075 Spain; 3 Biological Anthropology Unit, Department of Animal Biology, Plant Biology and Ecology, Faculty of Biosciences, Universitat Autònoma de Barcelona, Cerdanyola del Vallès, Barcelona, Catalonia 08193 Spain; 4 Institute for Interdisciplinary Studies, Faculty of Science, University of Amsterdam, Science Park 904, Amsterdam, 1098 XH The Netherlands; 5 Tissue Repair and Regeneration Laboratory (TR2Lab), Institut de Recerca i Innovació en Ciències de la Vida i de la Salut a la Catalunya Central (IrisCC), Ctra. de Roda, Vic, Barcelona, 08500 Spain; 6 Unitat Central de Balística i Traces Instrumentals, Mossos d’Esquadra, Av. de la Pau, 12, Sabadell, Barcelona, 08206 Spain; 7 Donor Center Barcelona Tissue Bank (BTB), Hospital Clínic de Barcelona, C/Villarroel 170, Escala 12 Planta 4, Barcelona, 08036 Spain; 8 Research Group of Biological Anthropology (GREAB), Biological Anthropology Unit, BABVE Department, Universitat Autònoma de Barcelona (UAB), Cerdanyola del Vallès, Bellaterra, Catalonia 08193 Spain

**Keywords:** Forensic anthropology, Human bones, Long bones, Femur, Gunshot trauma, Ballistic fracture pattern

## Abstract

Skeletal trauma assessment is an important task of forensic anthropologists and pathologists. This applies in particular to badly preserved bodies where the soft tissue cannot provide forensic evidence. Yet, the interpretation of ballistic long bone trauma can be difficult due to little conclusive data. Thus, this study explored the variability of diaphyseal fracture patterns dependent on the bullet’s angle and point of impact. 20 femurs from body donors were embedded in Clear Ballistics Gel^®^ and divided into 4 experimental groups: 70° angled shot on the centre of the anterior shaft aspect; perpendicular shot on the centre of the lateral shaft aspect; perpendicular shot on the centre of the posterior shaft aspect; grazing shot from posterior on the margin of the medial shaft aspect. In each case, a 9-mm Luger full metal jacket projectile was shot at a distance of 2 m and an impact speed of 360 m/s. All fractures were examined macroscopically. For the trauma comparison, a fifth group (perpendicular shot on the centre of the anterior shaft aspect), previously analysed in an earlier study, was included. Although the groups revealed similar fracture characteristics, the results suggest the bullet’s impact angle and location influence the fracture pattern. The most dissimilar fracture pattern was reproduced in the grazing shots, where only one defect hole was seen, instead of an entry and exit hole like in the other groups. The findings highlight the variability of ballistic fracture patterns in long bones and may serve as guidelines during the skeletal trauma assessment.

**Supplementary Information:**

The online version contains supplementary material available at 10.1007/s00414-025-03488-0.

## Introduction

In forensic anthropology and pathology, the investigation of gunshot trauma is a frequent and relevant challenge. Yet, the type of human tissue the specialists must deal with can vary fundamentally. In this context, the post-mortem examination of a well preserved body differs widely from that of a badly preserved one such as those that are severely decomposed, burnt, scavenged, mummified, saponified, mutilated or otherwise damaged [1]. In well preserved bodies, soft tissue injuries typically show characteristic features at first sight, permitting conclusions on the applied mechanical force [2]. The examination of soft tissue in ballistic wounds not only enables the differentiation from other type of trauma but yields further essential information for the event reconstruction [2]. Specifically, the wound characteristics usually allow the discrimination between bullet entrance and exit. Besides that, they can indicate further important clues such as information on the shooting distance or impact angle.

In badly preserved bodies, however, forensic evidence is often unattainable from the skin and soft tissue, making more resistant tissues like bone a crucial source of information. This resilience is due to bone’s unique structural composition, which includes a high mineral content alongside its organic component [3]. Although signs of trauma usually persist in bone, interpreting osseous trauma remains complex and challenging [4–6]. Particularly, when skeletal remains are damaged or incompletely recovered [7, 8].

The accurate and reliable interpretation of ballistic fractures is of great importance given the widespread use of firearms and the increasing numbers of gunshot victims on a global scale [9–12]. The literature further reveals that most of the gunshot injuries affect the bone [13]. However, compared to the well -established characteristics of soft tissue wounds, data on osseous gunshot trauma characteristics remains limited, particularly for bones other than the skull, such as long bones. In other words, while scientific studies on gunshot trauma have primarily focused on cranial fractures, injuries to long bones have received less attention [14–16]. This discrepancy may be explained by the extremely lethal nature of cranial gunshots, which aligns with the forensic pathologist’s primary task of determining the cause of death. Additionally, ballistic long bone trauma is inherently more difficult to analyse than cranial fractures due to the relatively small diameter of long bones resulting in small fragments with close proximity [17].

Nonetheless, the literature reveals that at least 50% of all gunshot injuries affect the extremities, often resulting in long bones fractures [13, 18–20]. In this regard, it has been claimed that the femoral shaft fracture is the most common injury among all ballistic long bone fractures. A recent review on ballistic long bone trauma identified various general fracture patterns, including linear, oblique, butterfly, and comminuted fractures [21]. While most studies have focused on these overall patterns, research on specific cortical traits remains limited, despite their potential relevance for the forensic analysis. Moreover, many forensic publications on this topic are case studies where key ballistic variables such as firearm type, ammunition, shooting distance, bullet impact angle and speed are unknown.

Experimental studies that allow for controlled analysis of such critical information are relatively rare, primarily due to ethical constraints and the complexities of handling human tissue in research. In a recent experimental study on human long bones, Schwab et al. identified a distinct ballistic fracture pattern in humeral and femoral shafts [22]. The authors comprehensively characterised cortical traits suggesting to be useful for the forensic trauma interpretation. In this context, they revealed that bullet entry and exit could be distinguished, allowing for the determination of shooting direction. However, those results were retrieved by testing one ballistic scenario. Given the dynamic nature of gunshot incidents, it is conceivable that fracture patterns may vary depending on different ballistic variables. Thus, more research is needed to gain more knowledge on gunshot fracture patterns. This study aims to test to which extent the diaphyseal fracture pattern changes under different shooting scenarios. In concrete, the influence of the bullet’s impact angle and impact location on the shaft was analysed based on four different experimental setups. The macroscopic fracture pattern was compared between those four groups and with the results from the previous study by Schwab et al. [22].

## Materials and methods

### Samples and sample preparation

20 fresh human femurs were exposed to experimental gunshot trauma. The bones were provided by the Donor Centre Barcelona Tissue Bank (Banc de Sang i Teixits de Catalunya) from 9 male and 3 female cadavers. Donor age at death was between 44 and 74 years.

The 20 bones were allocated into four shooting scenario groups: (1) 70° angled shot on the centre of the anterior shaft aspect; (2) perpendicular shot on the centre of the lateral shaft aspect; (3) perpendicular shot on the centre of the posterior shaft aspect; (4) grazing shot from posterior on the margin of the medial shaft aspect. The groups are further referred to as Anterior angled, Lateral, Posterior, and Grazing group, respectively.

In addition to those scenario groups, a fifth group of 10 human femurs analysed in a previous study by Schwab et al. [22] was used for trauma comparison. This group had been shot perpendicularly on the centre of the anterior shaft aspect, and is further referred to as Anterior group.

The sample preparation described in the following applies to all five sample groups. The femurs were removed from the limbs within 24 h post-mortem and stored at -80 °C until further processing. Before the shootings, the bones were thawed and the remaining soft tissue was carefully removed up to the periosteum using surgical tools. Each sample was visually examined to exclude potential damage or pathology. The bone length and anterior-posterior mid-shaft diameter were measured. The cortical bone thickness at the bullet impact was measured after the shootings. All sample characteristics are summarised in Table 1.

**Table 1:** Sample characteristics for all five groups, including sex, age, bone length, anterior-posterior (AP) shaft diameter, and cortical thickness (mean and standard deviation). Sample groups: anterior, anterior angled, lateral, posterior and grazing

Sample group	*N*	Female	Male	Age	Bone length (cm)	AP shaft diameter (mm)	Cortical thickness (mm)
Anterior	10	0	10	61 (5,4)	45,9 (2,8)	29,2 (2,5)	6,2 (0,8)
Anterior angled	5	1	4	68 (4,1)	44,6 (2,5)	30,8 (2,8)	7,8 (1,3)
Lateral	5	0	5	59 (14,3)	49,1 (3,3)	35,2 (2,5)	9,0 (1,2)
Posterior	5	2	3	60 (9,2)	45,2 (3,1)	31,2 (3,4)	9,8 (2,7)
Grazing	5	1	4	56 (11,9)	46,0 (4,1)	31,6 (3,2)	7,0 (2,0)

The femurs were placed in cylindrical moulds with a diameter of 10 cm. Before that, the target point was marked with a pen. To simulate soft tissue, the bones were surrounded with Clear Ballistics Gel^®^. This product is supplied as a ready mixed, solid block. In contrast to the 10% ordinal gelatine standard simulant, Clear Ballistics Gel^®^ is known for its exceptional transparency and clarity and its ease of preparation. The solid gel was melted for around 5 h at 100 °C before pouring it into the molds. It was then left to solidify overnight at ambient temperature. The resulting gel thickness at the target side was 2 cm.

### Experimental shooting conditions

To hold the femurs upright during the shootings, a self-constructed stabilisation device consisting of two metal plates connected by a steel bar was used [22, 23]. The lower plate was attached to a table for stability. The upper plate was adjustable for the different bone lengths. Metal cups were mounted on both plates, allowing the samples’ epiphyses to be firmly fixed with screws.

**Fig. 1:**
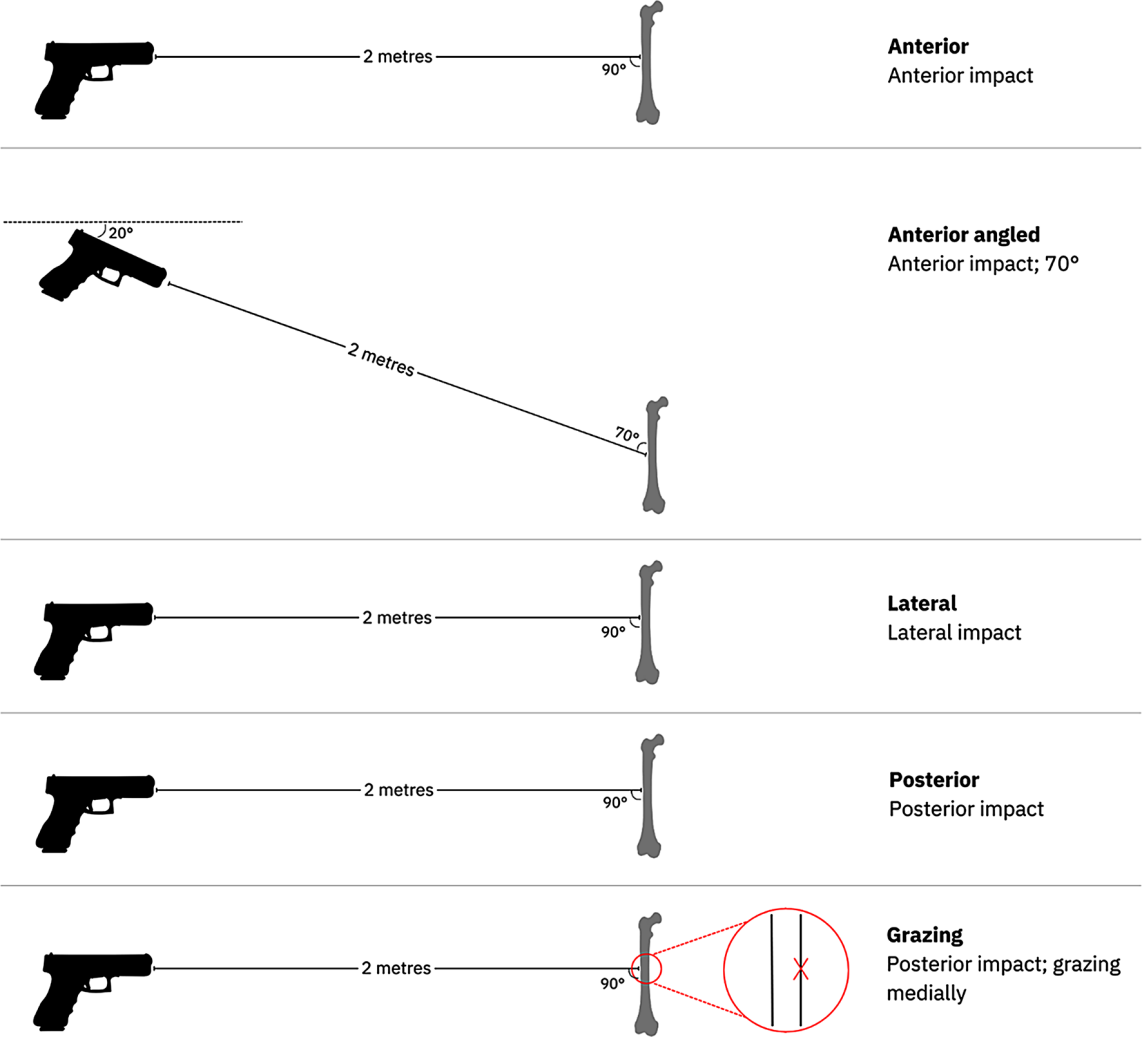
Schematic overview of all five shooting scenarios

All bones were shot from a distance of 2 m, using a 9 mm Luger test barrel (Drello Bal 1025 FU-R) and a NonTox 9 mm Luger full metal jacket projectile from Sellier & Bellot. The barrel was horizontally aligned, with exception for the Anterior angled group, where it was inclined 20° downwards. Figure 1 shows a simplified scheme of all five shooting scenarios.

### Sample assessment

After the gunshot, the bone fragments were carefully collected for each specimen. The epiphyses were removed with a bone saw. The samples were cleaned by boiling them at 100 °C for a maximum of 5 h in a water-detergent solution consisting of one cup of commercial degreasing detergent per 5 L of tap water. The next day we repeated the boiling process and removed the periosteum with a sponge [22–24]. After the samples were left to dry, we reassembled the fragments with a superglue.

For the qualitative analysis, the bones were assessed macroscopically to determine the fracture type, as well as the occurrence of entrance traits, exit traits and general cortical traits previously established by Schwab et al. [22]. The definitions of the traits are provided in the supplementary material (Online Resources 1a–c).

In addition, it was (re-)assessed whether the sample groups exhibited any other characteristics. Thus, the general trait “grey discolouration” around the bullet trajectory was included. Furthermore, for tip fragmentation, it was evaluated whether it appeared proximal and / or distal to the bullet entry. For the quantitative analysis, the maximal longitudinal fracture extent, as well as the horizontal and vertical diameter of the entry and exit holes were measured.

Chi-square was used to statistically test differences in the qualitative variables among groups. ANOVA or Kruskal-Wallis were applied to statistically test differences in the quantitative variables among groups. Pairwise comparisons were tested with Tukey post hoc. The significance level was set at 0,05. A multiple correspondence analysis (MCA) was performed to explore correlations between qualitative traits, bone properties (cortical thickness, bone length and shaft diameter), and sample groups.

### Ethics

This research followed the ethical precepts of the Declaration of Helsinki (Fortaleza, Brazil, Oct 2013). It was approved by the local ethics committee (Bellvitge University Hospital, L’Hospitalet de Llobregat, Barcelona, Spain; Ref. PR416/20). The human samples were processed according to the guidance for clinical use (EEC regulations 2004/23/CE and 2006/17/CE) and to the legal requirements of Spain (Law 14/2007, RD 1716/2011 and RD 9/2014). All human bones were donated anonymously and obtained under informed consent. All samples are stored in the private collection at the Institut de Medicina Legal i Ciències Forenses de Catalunya (IMLCFC) in Barcelona, Spain (Registro Nacional de Biobancos. Ref. C.0004241).

## Results

All shot samples displayed comminuted fractures, often accompanied by severe fragmentation. Piecing the larger fragments together allowed for the reconstruction of the fracture pattern and a sound macro-morphological evaluation. Most of the samples featured clearly distinguishable, separate bullet entry and exit holes, with the exception of the Grazing group, where the entry and exit appeared to have merged into one grazing hole. Entry and exit holes appeared mostly aligned. Only in the Anterior angled group, the exit hole was located more distal and, in some cases also slightly lateral, to the entry hole.

### Qualitative fracture characteristics

**Fig. 2:**
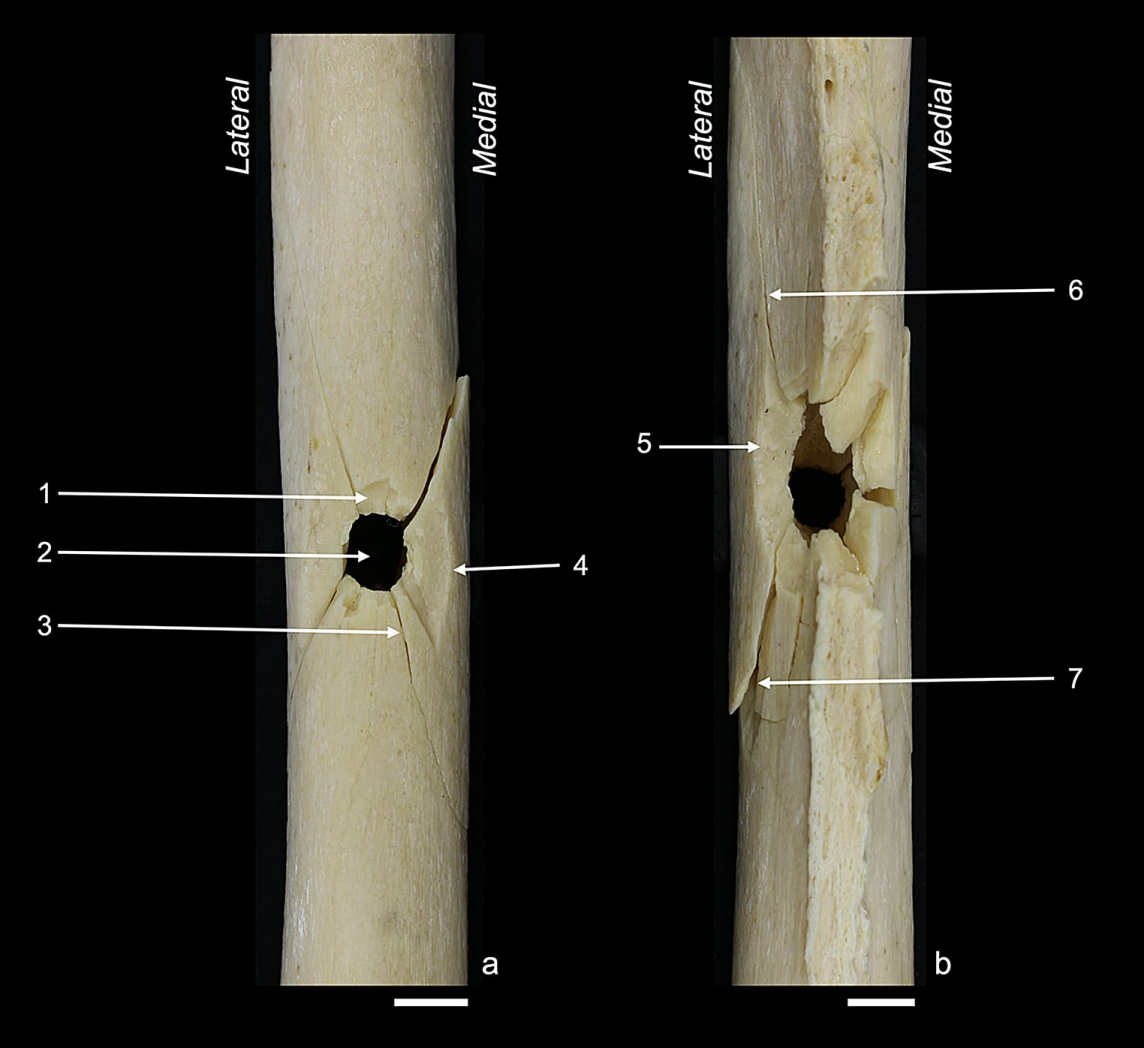
Anterior sample: (**a**) anterior view showing the bullet entry; (**b**) posterior view showing the bullet exit. Labelled traits: (1) tip fragmentation; (2) round entry hole; (3) entry-associated radiating fracture; (4) wing piece with wing flake defect; (5) fracture surface scaling; (6) exit-associated radiating fracture; (7) plastic deformation. White bar = 1 cm

**Fig. 3:**
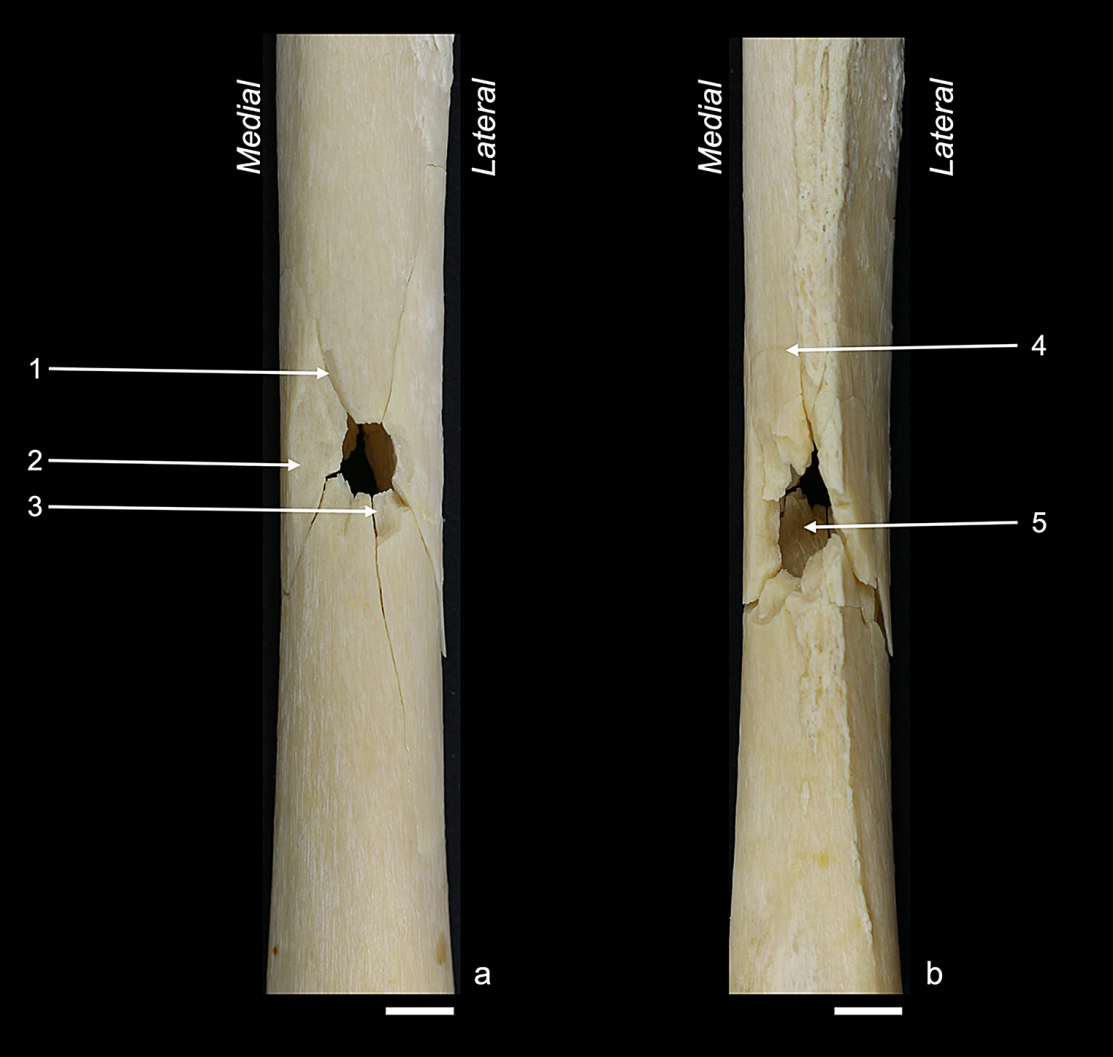
Anterior angled sample: (**a**) anterior view showing the bullet entry; (**b**) posterior view showing the bullet exit. Labelled traits: (1) marginal chipping; (2) wing flake defect; (3) tip fragmentation distal to the entry; (4) exit-associated transversal concentric fracture; (5) square exit hole, laterally deviated. White bar = 1 cm

**Fig. 4:**
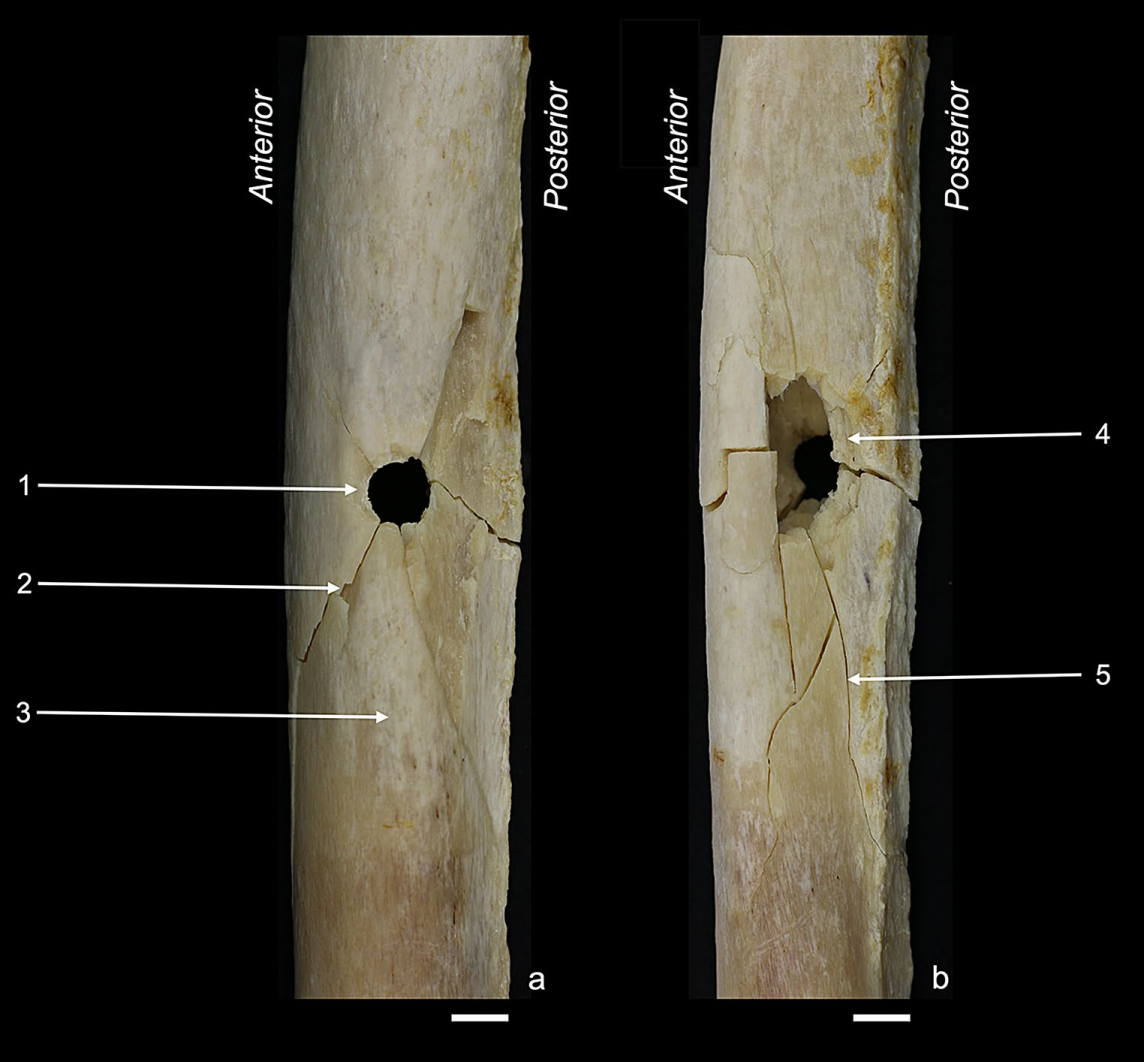
Lateral sample: (**a**) lateral view showing the bullet entry; (**b**) medial view showing the bullet exit. Labelled traits: (1) ring defect; (2) marginal chipping; (3) v-shape; (4) external bevelling; (5) exit-associated radiating fracture. White bar = 1 cm

**Fig. 5:**
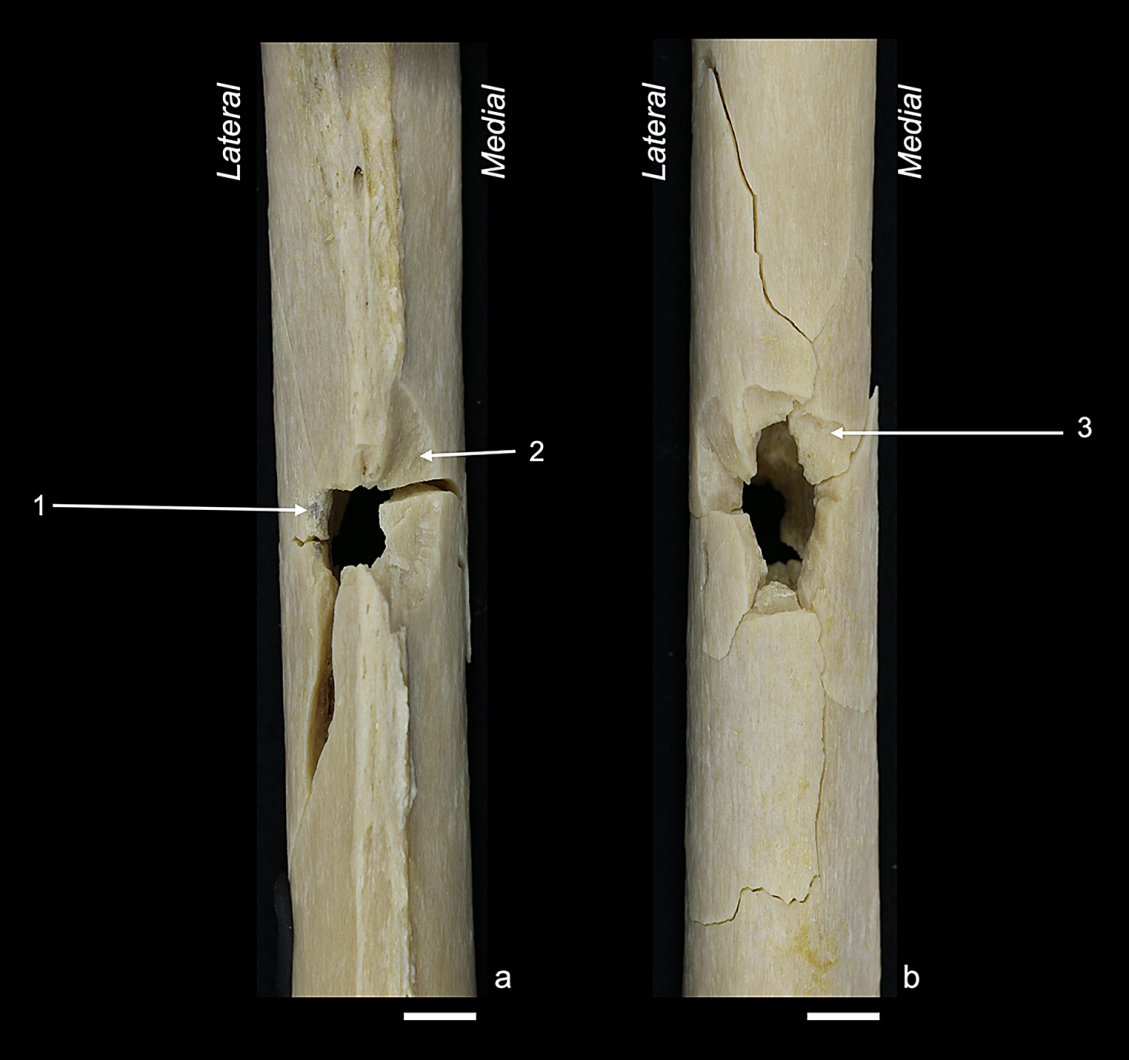
Posterior sample: (**a**) posterior view showing the bullet entry; (**b**) anterior view showing the bullet exit. Labelled traits: (1) grey discolouration; (2) wing flake defect; (3) external bevelling. White bar = 1 cm

**Fig. 6:**
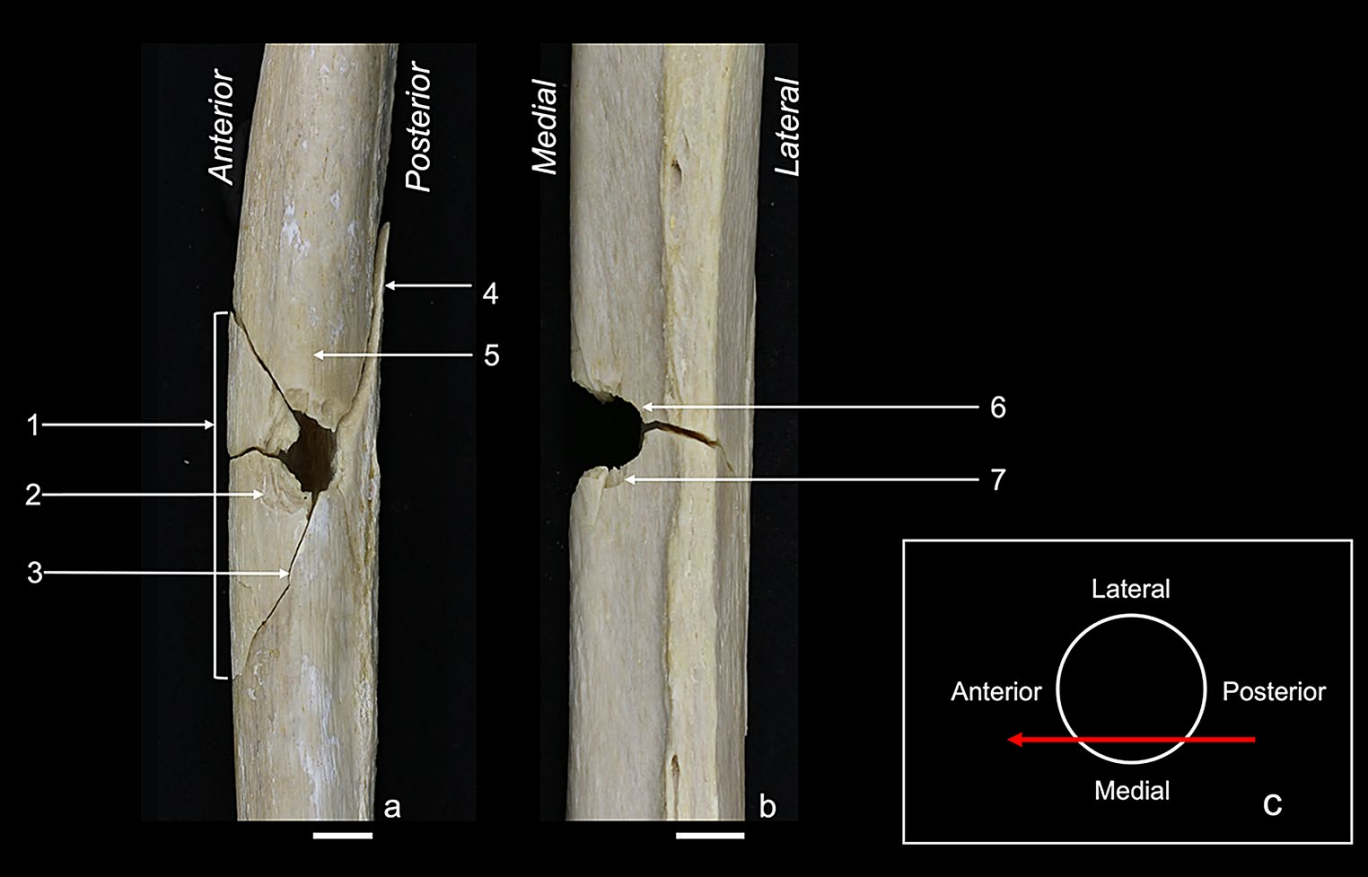
Grazing samples: (**a**) medial view showing the grazing hole; (**b**) posterior view showing the bullet entry side; (**c**) Schematic representation of a transversal shaft view, showing the bullet trajectory (red arrow). Labelled traits: (1) wing piece, transversally broken; (2) superficial cortical damage at the bullet exit; (3) exit-associated radiating fracture; (4) entry-associated radiating fracture and plastic deformation; (5) v-shape; (6) round entry hole; (7) tip fragmentation. White bar = 1 cm

Figures 2, 3, 4, 5 and 6 illustrate the fracture patterns of the different sample groups. The respective occurrence values of all 28 cortical traits are listed in Table 2. The majority of the cortical traits that had been characterised by Schwab et al. [22] for perpendicular anterior shots was also found in the other sample groups of this study.

**Table 2:** Occurrence of the cortical traits for each sample group (* *p*-value < 0,05; ** *p*-value < 0,001)

	Anterior		Anterior angled		Lateral		Posterior		Grazing	
*N*	10		5		5		5		5	
***Entrance traits***										
Round hole	10	100%	5	100%	5	100%	5	100%	5	100%
Radiating fracture	10	100%	5	100%	5	100%	5	100%	5	100%
Concentric fracture*	10	100%	4	80%	3	60%	2	40%	0	0%
longitudinal*	10	100%	4	80%	3	60%	2	40%	0	0%
transversal	0	0%	0	0%	0	0%	1	20%	0	0%
V-shape*	10	100%	4	80%	5	100%	3	60%	2	40%
Ring defect	10	100%	5	100%	5	100%	5	100%	4	80%
Tip fragmentation*	9	90%	5	100%	5	100%	5	100%	2	40%
proximal*	8	80%	0	0%	5	100%	3	60%	2	40%
distal*	9	90%	5	100%	2	40%	4	80%	1	20%
Wing flake	6	60%	4	80%	3	60%	1	20%	0	0%
Wing flake defect*	9	90%	4	80%	3	60%	2	40%	0	0%
Wing piece	10	100%	4	80%	5	100%	5	100%	4	80%
Lateral notch	10	100%	3	60%	3	60%	4	80%	3	60%
Internal bevelling*	10	100%	5	100%	5	100%	3	60%	5	100%
***Exit traits***										
Square hole	9	90%	4	80%	2	40%	2	40%	N/A	
Radiating fracture	10	100%	5	100%	5	100%	5	100%	5	100%
Concentric fracture	10	100%	5	100%	5	100%	5	100%	4	80%
longitudinal*	7	70%	1	20%	3	60%	5	100%	5	100%
transversal**	10	100%	5	100%	5	100%	4	80%	0	0%
Layered breakage	9	90%	5	100%	3	60%	5	100%	4	80%
External bevelling**	10	100%	5	100%	5	100%	4	80%	0	0%
Stepped breakout*	8	80%	2	40%	2	40%	1	20%	0	0%
***General traits***										
Plastic deformation	10	100%	5	100%	5	100%	5	100%	5	100%
Marginal chipping	10	100%	5	100%	5	100%	5	100%	5	100%
Fracture surface scaling	10	100%	5	100%	5	100%	5	100%	5	100%
Grey discolouration*	4	40%	0	0%	3	60%	5	100%	3	60%

### Entrance fracture characteristics

In every instance, the entrance fracture displayed a round entry hole with radiating fractures, visually resulting in a stellate pattern. Concentric fractures were present in all Anterior samples (100%), in four of five Anterior angled samples (80%), in three of five Lateral samples (60%), in two of five Posterior samples (40%), but in no Grazing sample (0%). The difference between the groups was statistically significant ($\:{\chi\:}^{2}$ = 16,2; df = 4; *p* = 0,003). The concentric fractures were divided into fractures arranged longitudinal and transversal to the shaft. Longitudinal concentric fractures were present in all Anterior samples (100%), in four of five Anterior angled samples (80%), in three of five Lateral samples (60%), in two of five Posterior samples (40%), but in no Grazing sample (0%). The difference between the groups was statistically significant ($\:{\chi\:}^{2}$ = 16,2; df = 4; *p* = 0,003). Transversal concentric fractures were exclusively present in one of five Posterior samples (20%). They did not occur in the other groups (0% each). This difference was not statistically significant ($\:{\chi\:}^{2}$ = 5,17; df = 4; *p* = 0,27).

V-shape was observed in all Anterior and Lateral samples (100% each), in four of five Anterior angled samples (80%), in three of five Posterior samples (60%), and in two of five Grazing samples (40%). The difference between the groups was statistically significant ($\:{\chi\:}^{2}$ = 10,0; df = 4; *p* = 0,04).

Ring defect was present in all samples of the Anterior, Anterior angled, Lateral, and Posterior group (100% each), and in four of five Grazing samples (80%). This difference was not statistically significant ($\:{\chi\:}^{2}$ = 5,17; df = 4; *p* = 0,27). However, in the Anterior angled group, the ring defect proximal to the entrance hole was less pronounced. Similarly, the Grazing samples also presented a less pronounced ring defect.

Tip fragmentation was present in all Anterior angled, Lateral, and Posterior samples (100% each), in nine of ten Anterior samples (90%), and in two of five Grazing samples (40%). The difference between the groups was statistically significant ($\:{\chi\:}^{2}$ = 11,8; df = 4; *p* = 0,019). Tip fragmentation proximal to the entry hole was seen in all Lateral samples (100%), in eight of ten Anterior samples (80%), in three of five Posterior samples (60%), in two of five Grazing samples (40%), but in no Anterior angled sample (0%). The difference between the groups was statistically significant ($\:{\chi\:}^{2}$ = 13,3; df = 4; *p* = 0,01). Tip fragmentation distal to the entry hole was seen in all Anterior angled samples (100%), in nine of ten Anterior samples (90%), in four of five Posterior samples (80%), in two of five Lateral samples (40%), and in one of five Grazing samples (20%). The difference between the groups was statistically significant ($\:{\chi\:}^{2}$ = 12,4; df = 4; *p* = 0,015).

Wing flake was found in four of five Anterior angled samples (80%), in six of ten Anterior samples (60%), in three of five Lateral samples (60%), in one of five Posterior samples (20%), but in no Grazing sample (0%). The difference was close to statistically significant ($\:{\chi\:}^{2}$ = 9,11; df = 4; *p* = 0,058).

Wing flake defect was observed in nine of ten Anterior samples (90%), in four of five Anterior angled samples (80%), in three of five Lateral samples (60%), in two of five Posterior samples (40%), but in no Grazing sample (0%). The difference between the groups was statistically significant ($\:{\chi\:}^{2}$ = 12,9; df = 4; *p* = 0,012).

Wing piece was present in all Anterior, Lateral, and Posterior samples (100% each), and in four of five Anterior angled and Grazing samples (80% each). The difference was not statistically significant ($\:{\chi\:}^{2}$ = 4,29; df = 4; *p* = 0,369). Particular note, wing piece was not exclusively formed by entry-associated fractures, but also by exit-associated fractures.

Lateral notch was observed in all Anterior samples (100%), in four of five Posterior samples (80%), and in three of five Anterior angled, Lateral, and Grazing samples (60% each). The difference was not statistically significant ($\:{\chi\:}^{2}$ = 5,4; df = 4; *p* = 0,248).

**Fig. 7:**
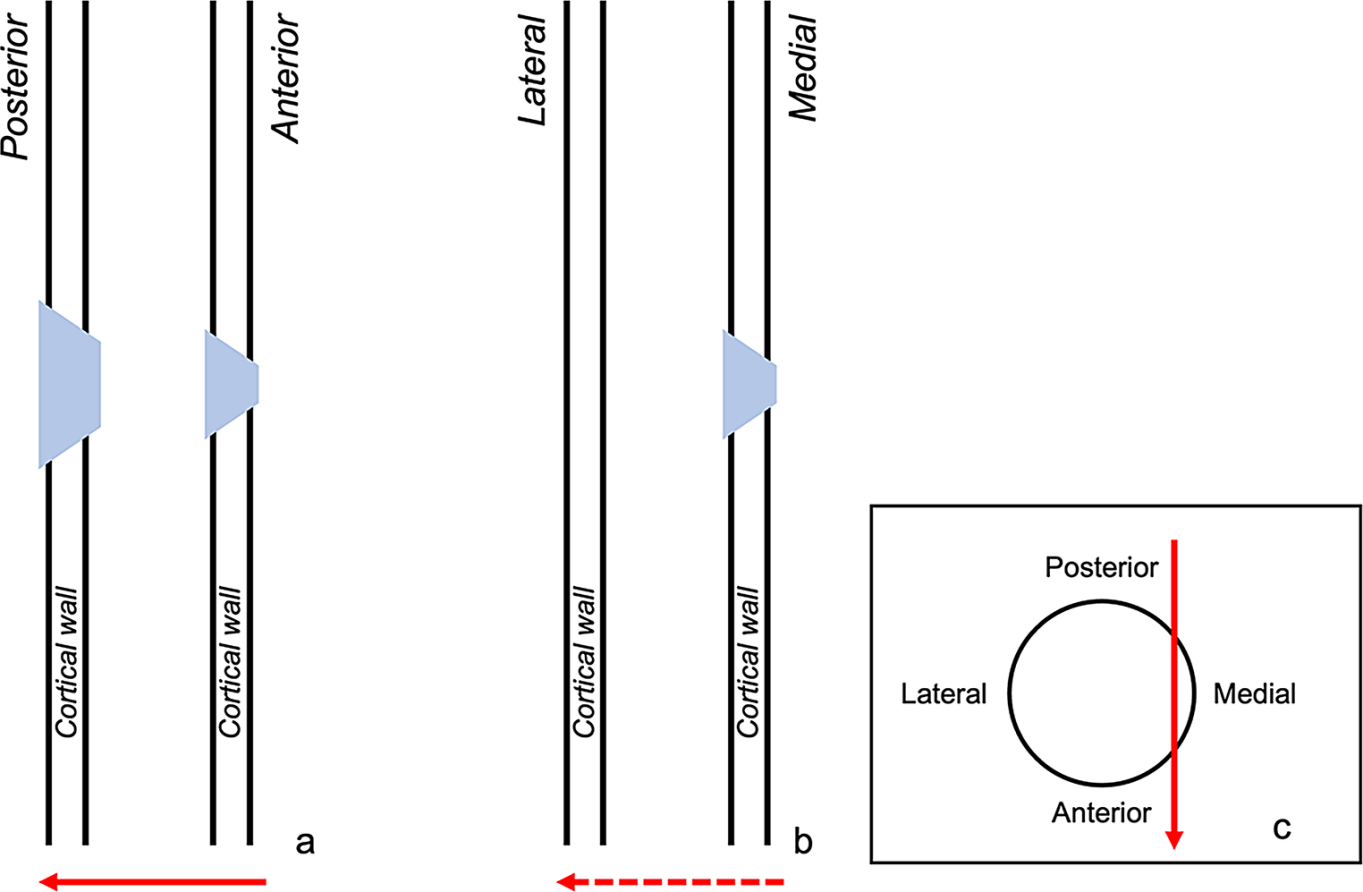
Schematic illustrations: (**a**) Anterior shot from a lateral view on the shaft showing internal and external bevelling (small and big blue funnel, respectively) leading to a correct interpretation of the bullet’s flight direction from anterior to posterior (red arrow); (**b**) Grazing shot from an anterior view on the shaft with internal bevelling (blue funnel) leading to an incorrect interpretation of the bullet’s flight direction from medial to lateral (dashed red arrow); (**c**) Grazing shot from a transversal view of the shaft, showing the correct flight direction from posterior to anterior (red arrow)

Internal bevelling was present in all Anterior, Anterior angled, Lateral, and Grazing samples (100% each). In the Posterior group it was featured in three of five samples (60%). The difference was statistically significant ($\:{\chi\:}^{2}$ = 10,7; df = 4; *p* = 0,03). Particular note, in the Grazing samples internal bevelling did not indicate the correct bullet direction. The greater extent of the funnel pointed laterally towards the interior of the shaft, and not anteriorly towards the bullet exit (Fig. 7).

### Exit fracture characteristics

Square exit hole was observed in nine of ten Anterior samples (90%), in four of five Anterior angled samples (80%) and in two of five Lateral and Posterior samples (40% each). The other samples in these groups featured a rounded exit hole. The difference between these groups was not statistically significant ($\:{\chi\:}^{2}$ = 6,16; df = 3; *p* = 0,104). The Grazing group was excluded for the statistical analysis of this trait, because the tangential exit defect did not allow to draw conclusions on the shape.

All samples exhibited radiating fractures (100% each). Concentric fractures were present in all Anterior, Anterior angled, Lateral and Posterior samples (100% each), and in four of five Grazing samples (80%). The difference was not statistically significant ($\:{\chi\:}^{2}$ = 5,17; df = 4; *p* = 0,27). Longitudinal concentric fractures were observed in all Posterior and Grazing samples (100% each), in seven of ten Anterior samples (70%), in three of five Lateral samples (60%), and in one of five Anterior angled samples (20%). The difference between the groups was statistically significant ($\:{\chi\:}^{2}$ = 10,5; df = 4; *p* = 0,033). Transversal concentric fractures were present in all Anterior, Anterior angled and Lateral samples (100% each), in four of five Posterior samples (80%), but in no Grazing sample (0%). The difference between the groups was statistically significant ($\:{\chi\:}^{2}$ = 25; df = 4; *p* = < 0,001).

Layered breakage was observed in all Anterior angled and Posterior samples (100% each), in nine of ten Anterior samples (90%), in three of five Lateral samples (60%) and in four of five Grazing samples (80%). The difference was not statistically significant ($\:{\chi\:}^{2}$ = 4,9; df = 4; *p* = 0,297).

External bevelling was present in all Anterior, Anterior angled, and Lateral samples (100% each), in four of five Posterior samples (80%), but in no Grazing sample (0%). The difference between the groups was statistically significant ($\:{\chi\:}^{2}$ = 25; df = 4; *p* = < 0,001).

Stepped breakout was observed in eight of ten Anterior samples (80%), in two of five Anterior angled and Lateral samples (40% each), in one of five Posterior samples (20%), but in no Grazing sample (0%). The difference was statistically significant ($\:{\chi\:}^{2}$ = 10,5; df = 4; *p* = 0,033).

### General fracture characteristics

All samples featured plastic deformation, marginal chipping and fracture surface scaling (100% each). Grey discolouration was observed in all Posterior samples (100%), in three of five Lateral and Grazing samples (60% each), in four of ten Anterior samples (40%), but in no Anterior angled sample (0%). The difference was statistically significant ($\:{\chi\:}^{2}$ = 10,8; df = 4; *p* = 0,029).

### Quantitative fracture characteristics

**Fig. 8:**
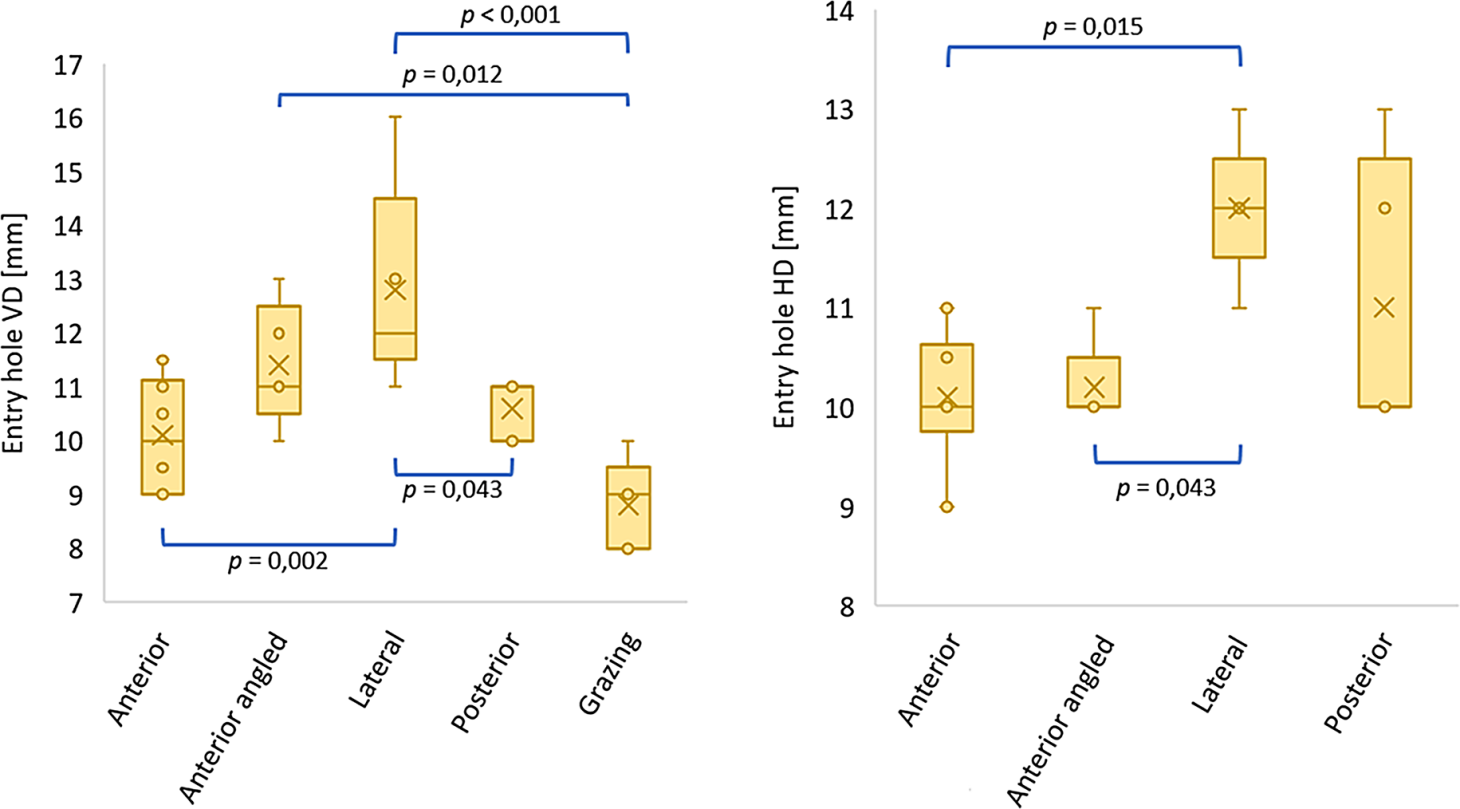
Boxplots representing the entry holes’ vertical diameter (VD) and horizontal diameter (HD) for each evaluated sample group (significant *p*-values for the pairwise comparison)

**Fig. 9:**
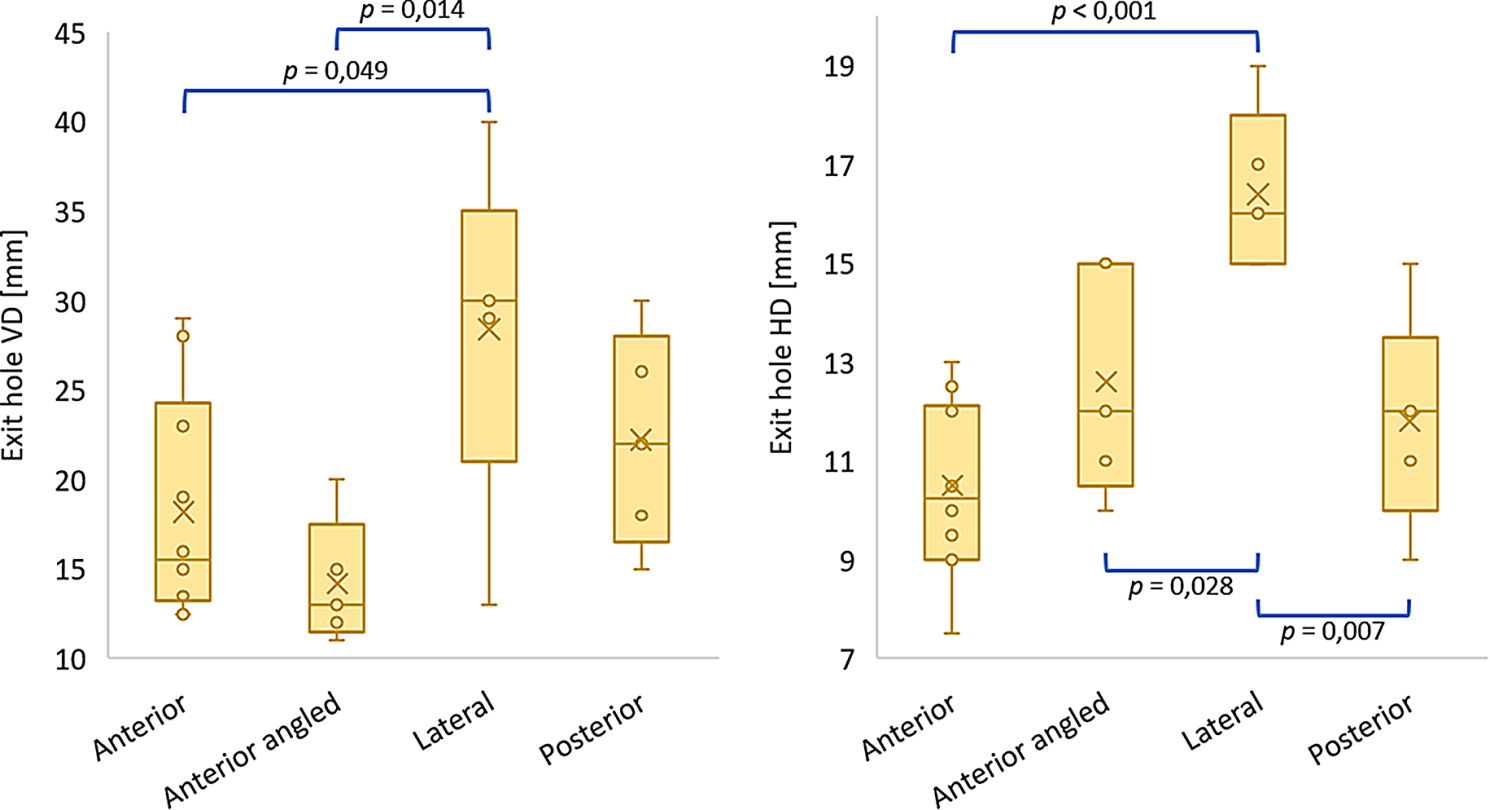
Boxplots representing the exit holes’ vertical diameter (VD) and horizontal diameter (HD) for each evaluated sample group (significant *p*-values for the pairwise comparison)

**Fig. 10:**
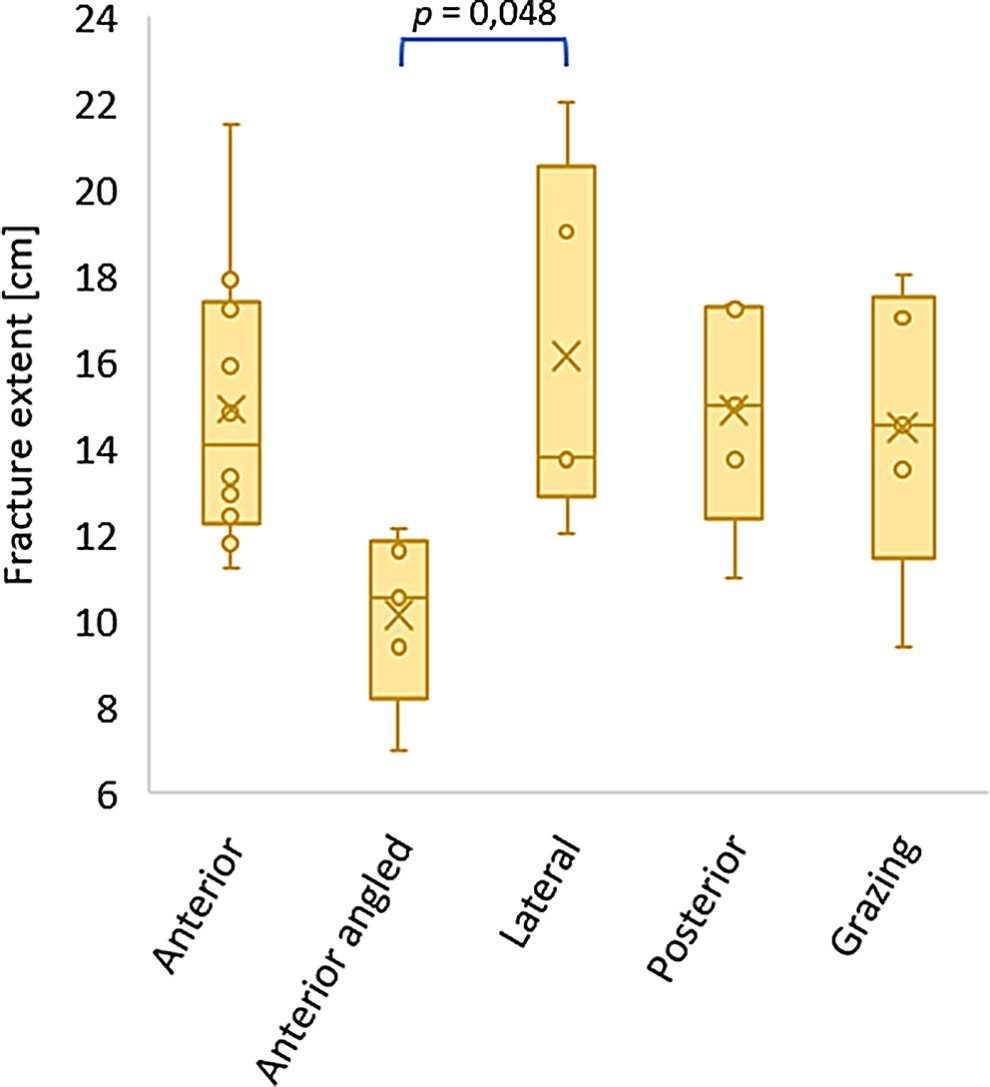
Boxplots representing the fracture extent for each sample group (significant *p*-value for the pairwise comparison)

All values of the quantitative variables are listed in Table 3. Figures 8, 9 and 10 display the boxplots and significant *p*-values for the pairwise comparison of each quantitative variable. With regard to the entry and exit hole size, the Grazing group only allowed to measure the vertical entry hole diameter.

**Table 3:** Descriptive statistics of the analysed quantitative traits (* *p*-value < 0,05; ** *p*-value < 0,001). VD, vertical diameter; HD, horizontal diameter

	Group	*N*	Mean	SD	Min	Max
Entry VD (mm)*	Anterior	10	10,1	1,02	9,0	11,5
	Anterior angled	5	11,4	1,14	10,0	13,0
	Lateral	5	12,8	1,92	11,0	16,0
	Posterior	5	10,6	0,55	10,0	11,0
	Grazing	5	8,8	0,84	8,0	10,0
Entry HD (mm)*	Anterior	10	10,1	0,69	9,0	11,0
	Anterior angled	5	10,2	0,45	10,0	11,0
	Lateral	5	12,0	0,71	11,0	13,0
	Posterior	5	11,0	1,41	10,0	13,0
Exit VD (mm)*	Anterior	10	18,2	6,33	12,5	29,0
	Anterior angled	5	14,2	3,56	11,0	20,0
	Lateral	5	28,4	9,71	13,0	40,0
	Posterior	5	22,2	6,02	15,0	30,0
Exit HD (mm)*	Anterior	10	10,5	1,81	7,5	13,0
	Anterior angled	5	12,6	2,30	10,0	15,0
	Lateral	5	16,4	1,67	15,0	19,0
	Posterior	5	11,8	2,17	9,0	15,0
Fracture extent (cm)*	Anterior	10	14,9	3,25	11,2	21,5
	Anterior angled	5	10,1	2,03	7,0	12,1
	Lateral	5	16,1	4,22	12,0	22,0
	Posterior	5	14,8	2,63	11,0	17,3
	Grazing	5	14,5	3,37	9,4	18,0

Compared to the projectile’s diameter, the mean vertical entry hole diameter was larger in all sample groups with exception of the Grazing group, where it was smaller. The largest mean vertical entry hole diameter was found in the Lateral group, followed by the Anterior angled, Posterior, Anterior and Grazing group, respectively. One-way ANOVA showed significant differences between the groups (*F =* 6,54; df = 4–10,7; *p* = 0,006). Pairwise comparisons showed a significant difference between the Lateral and Anterior group, the Lateral and Posterior group, the Lateral and Grazing group, and the Anterior angled and Grazing group (Fig. 8).

Compared to the projectile’s diameter, the mean horizontal entry hole diameter was in all evaluable sample groups larger. The mean horizontal entry hole diameter was the largest in the Lateral group, followed by the Posterior, Anterior angled and Anterior group, respectively. Kruskal-Wallis showed significant differences between the groups ($\:{\chi\:}^{2}$ = 10.1; df = 3; *p* = 0.018). Pairwise comparisons revealed a significant difference between the Lateral and Anterior group, and the Lateral and Anterior angled group (Fig. 8).

The mean vertical exit hole diameter was the largest in the Lateral group, followed by the Posterior, Anterior and Anterior angled group, respectively. One-way ANOVA revealed significant differences between the groups (*F* = 4,03; df = 3–9,66; *p* = 0,042). Pairwise comparisons showed a significant difference between the Lateral and Anterior angled group, and the Lateral and Anterior group (Fig. 9).

The mean horizontal exit hole diameter was the largest in the Lateral group, followed by the Anterior angled, Posterior and Anterior group, respectively. One-way ANOVA revealed significant differences between the groups (*F* = 11,6; df = 3–9,35; *p* = 0,002). Pairwise comparisons showed a significant difference between the Lateral and Anterior group, the Lateral and Anterior angled group, and the Lateral and Posterior group (Fig. 9).

The mean fracture extent was the largest in the Lateral group, followed by the Anterior, Posterior, Grazing and Anterior angled group, respectively. One-way ANOVA showed significant differences between the groups (*F* = 4,05; df = 4–10,9; *p* = 0,03). Pairwise comparisons showed a significant difference between the Anterior angled and Lateral group (Fig. 10).

### Multiple correspondence analysis (MCA)

**Fig. 11:**
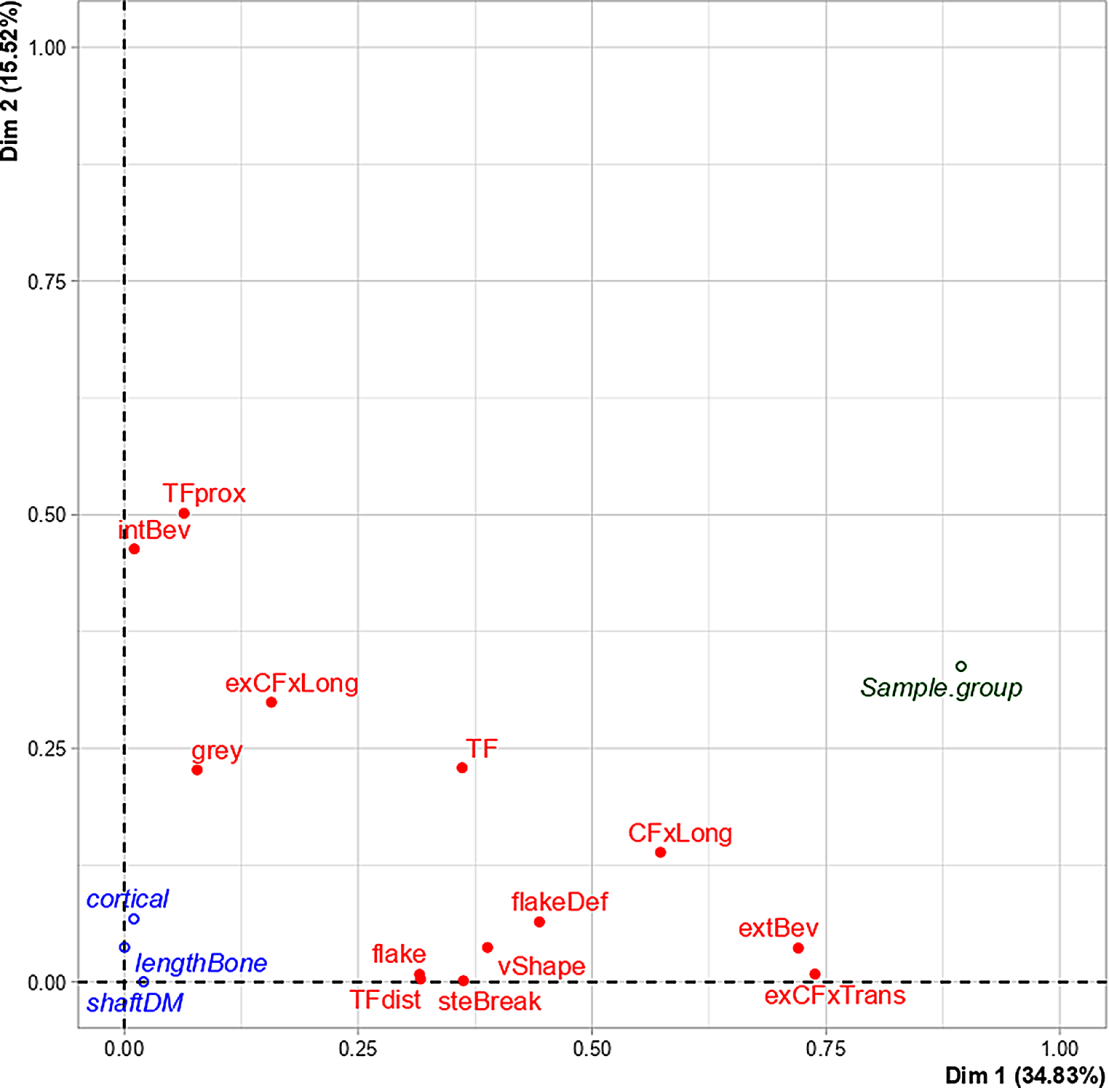
Multiple correspondence analysis’s biplot of variables (qualitative traits, sample groups, bone properties) representing the two dimensions (Dim 1 and Dim 2) with the associated variance in brackets. Representation of the traits: *TF*– tip fragmentation; *TFprox*– tip fragmentation proximal to the entry; *TFdist*– tip fragmentation distal to the entry; *intBev*– internal bevelling; *extBev*– external bevelling; *exCFxTrans*– transversal concentric fracture exit; *exCFxLong*– longitudinal concentric fracture exit; *grey*– grey discolouration; *CFxLong*– longitudinal concentric fracture entry; *flake*– wing flake; *flakeDef*– wing flake defect; *vShape*– V-shape; *steBreak*– stepped breakout; *cortical*– cortical thickness; *lengthBone*– bone length; *shaftDM*– AP shaft diameter

**Fig. 12:**
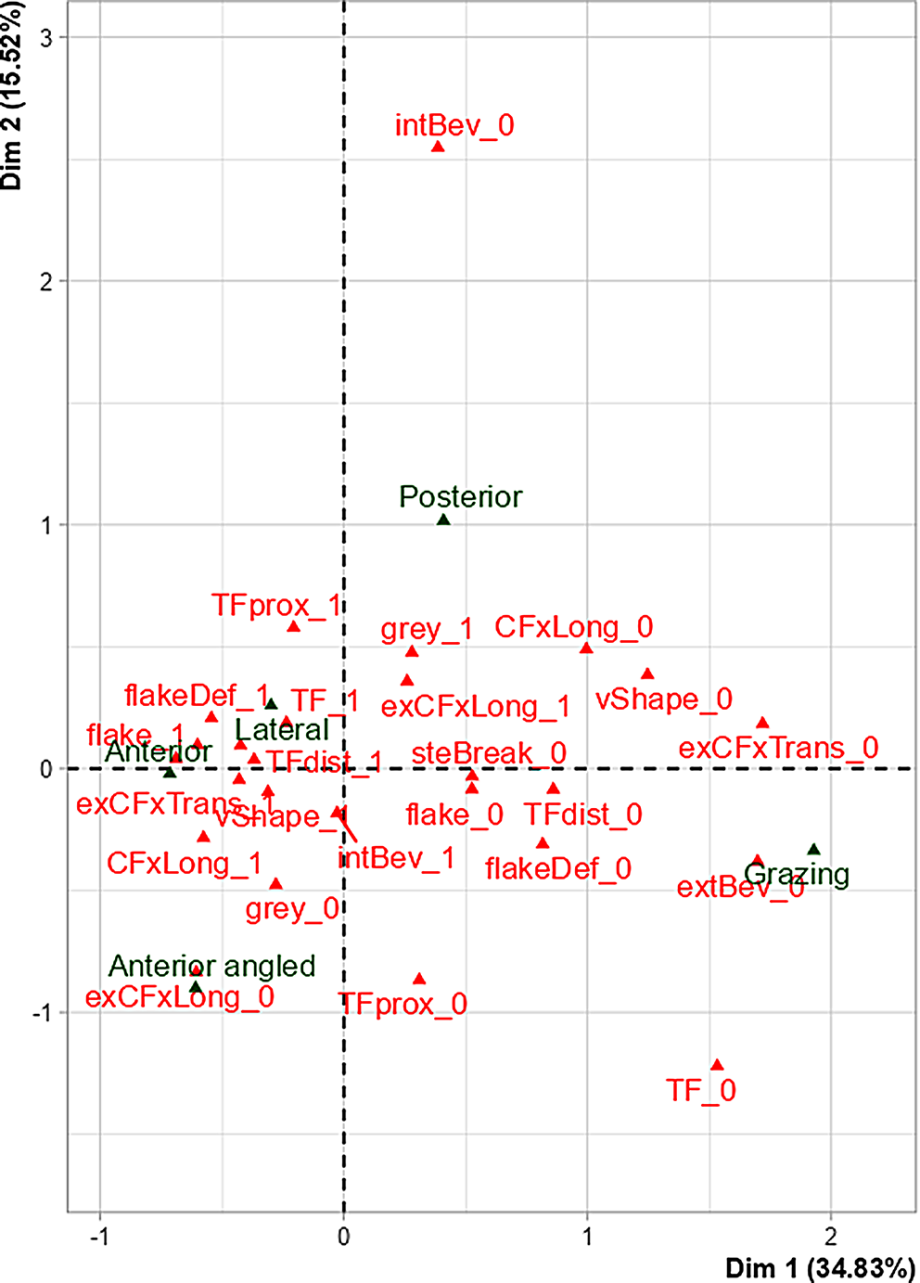
Multiple correspondence analysis’s biplot of categories within the variables (qualitative traits and sample groups) representing Dim 1 and Dim 2 with their associated variance in brackets. Representation of the traits (1, present; 0, absent): *TF*– tip fragmentation; *TFprox*– tip fragmentation proximal of the entry; *TFdist*– tip fragmentation distal of the entry; *intBev*– internal bevelling; *extBev*– external bevelling; *exCFxTrans*– transversal concentric fracture exit; *exCFxLong*– longitudinal concentric fracture exit; *grey*– grey discolouration; *CFxLong*– longitudinal concentric fracture entry; *flake*– wing flake; *flakeDef*– wing flake defect; *vShape*– V-shape; *steBreak*– stepped breakout

An MCA was performed to explore correlations between qualitative fracture traits, bone properties and sample groups. With regard to the qualitative traits, in total thirteen were included based on their statistical significance in the bivariate analysis. Table 4; Figs. 11 and 12 represent the first two dimensions explaining together 50,35% of the total variance.

Dimension 1 shows a correlation between the sample groups and exit-associated transversal concentric fractures, external bevelling, entry-associated longitudinal concentric fractures, flake defect, v-shape, stepped breakout, (distal) tip fragmentation, wing flake, and exit-associated longitudinal concentric fracture (Table 4; Fig. 11).

**Table 4:** Description of the multiple correspondence analysis’s axes. Significant correlations of the variables with the first two dimensions are shown. Category: (-) trait absence, (+) trait presence. Abbreviations of the variables: *exCFxTrans–* transversal concentric fracture exit; *extBev*– external bevelling; *CFxLong*– longitudinal concentric fracture entry; *flakeDef*– wing flake defect; *vShape*– V-shape; *steBreak*– stepped breakout; *TF*– tip fragmentation; *TFdist*– tip fragmentation distal to the entry; *flake*– wing flake; *exCFxLong*– longitudinal concentric fractures exit; *TFprox*– tip fragmentation proximal to the entry; *intBev*– internal bevelling; *grey*– grey discolouration

Percentage of the variance	Variable	Category	R2	*p*-value
Dim 1 (34,83%)	Sample group	Grazing	0,8944	7,656e-12
	exCFxTrans	-	0,7382	1,224e-09
	extBev	-	0,7203	3,111e-09
	CFxLong	-	0,5730	1,290e-06
	flakeDef	-	0,4436	5,879e-05
	vShape	-	0,3881	2,358e-04
	steBreak	-	0,3624	4,327e-04
	TF	-	0,3611	4,460e-04
	TFdist	-	0,3164	1,213e-03
	flake	-	0,3156	1,237e-03
	exCFxLong	+	0,1572	3,010e-02
Dim 2 (15,52%)	TFprox	+	0.5015	1.198e-05
	intBev	-	0.4634	3.478e-05
	exCFxLong	+	0.2993	1.756e-03
	TF	+	0.2292	7.445e-03
	grey	+	0.2270	7.774e-03
	Sample group	Posterior	0.3377	3.026e-02
	CFxLong	-	0.1387	4.265e-02

Dimension 2 shows a correlation between the Posterior group and the presence of (proximal) tip fragmentation, exit-associated longitudinal concentric fracture and grey discolouration as well as the absence of internal bevelling and entry-associated longitudinal concentric fracture (Table 4).

Figure 12 shows that the Anterior and Lateral group presented the most similar occurrence of most of the traits. Figure 12 also shows that the Posterior group differed from the Anterior and Lateral group particularly by a lower occurrence of internal bevelling. The Anterior angled group differed from the Anterior and Lateral group particularly by a lower occurrence of exit-associated longitudinal concentric fracture. Furthermore, Fig. 12 illustrates that the Grazing group differed the most from the other groups. This was characterised by a lower occurrence of most of the traits: most specifically, external bevelling, exit-associated transversal concentric fracture, flake defect and entry-associated longitudinal concentric fracture.

Moreover, the results of the MCA revealed that bone properties such as cortical thickness, bone length and shaft diameter did not significantly influence the occurrence of the cortical traits (Fig. 11).

## Discussion

Understanding differences in the appearance of ballistic fractures is crucial due to the inherent dynamic of gunshot incidents. In a previous approach, Schwab et al. [22] explored the fracture pattern in human long bones by testing one shooting scenario: An orthograde shot towards the anterior shaft centre with a 9 mm Luger FMJ projectile at an impact speed of 360 m/s and a distance of 2 m. As a continuation, the present study examined the variability of the established fracture pattern by analysing four additional shooting scenarios: an orthograde shot towards the lateral and posterior shaft centre, a 70° angled shot towards the anterior shaft centre and a grazing shot from posterior towards the medial shaft margin. Hence, this exclusive work provides further insight into the ballistic fracture pattern in human long bones, incorporating alterations of the bullet impact location and angle.

### Similarity of the fracture patterns

As frequently reported, all femurs shattered upon gunshot, resulting in comminuted fractures [21]. Particularly the fractures of the Anterior, Anterior angled, Lateral and Posterior group appeared to be quite similar at first sight. They all presented with two main defects on the shaft: the bullet entry and exit. In contrast, the Grazing group appeared somewhat different with only one main defect: a merged bullet entry and exit. With a few reservations mainly in the Grazing group, the previously established cortical traits by Schwab et al. [22] were largely reproducible in all sample groups. This indicates that the revealed fracture pattern can also be reproduced when the impact location and angle change in diaphyseal shots. Additionally, this study revealed a further trait named grey discolouration. Although its appearance might resembles a bullet wipe, defined as a grey circle around the entry hole [23], it was not classified as such because the grey pigments were not circular and not limited to the entry hole. Instead, they also appeared in the exit area. Further research, including detailed chemical analyses, may help clarify the origin of these pigments. Moreover, increasing the sample size would enable a more thorough assessment of the consistency of this phenomenon across different cases.

With respect to the trait’s occurrence values, the Anterior and Lateral group featured the most similar fracture pattern. In comparison, the Grazing group differed the most from the others. All differences in the trait’s occurrence values will be discussed below for each group. Before that, the traits with no significant differences among the groups will be considered. The bullet entry consistently appeared as a round hole. This has previously been reported in perpendicular impacts [17, 25]. This study further suggests that an angled shot from above also produces a round defect. With respect to caliber estimation, caution is advised when inferring bullet size from entry hole diameters. In cases where an entire entry hole was present, the measured values consistently exceeded the actual bullet diameter, aligning with observations made in cranial entry wounds [26–28]. Across all sample groups, radiating fractures ran away from the bullet entry, typically forming a stellate pattern, consistent with previous research [16, 17]. Nonetheless, only part of this stellate pattern can be seen in the Grazing group, due to the nature of the tangential impact. Ring defect was revealed to be a shared characteristic in all groups. Similar findings have been reported for cranial gunshots [29–32]. Wing piece and lateral notch were also reproducible to a similar degree in all groups. Lately, the first has been described as a fragment that is defined by two entry-associated radiating fractures and one entry-associated concentric fracture [22, 23]. This study, however, revealed that wing piece can also include exit-associated fractures. Thus, we recommend to identify this trait by its characteristic shape and not by the classification of the underlying fracture lines. Another common feature was that transversal concentric fractures were very unlikely to form around the bullet entry (with only one single case in the Posterior group).

The exit hole was typically larger than the entry hole, consistent with other studies that report more destructive exits than entries [7, 33, 34]. In all groups, the bullet exit caused radiating and concentric fractures. Layered breakage was also observed across all groups. However, it is not unique to gunshot trauma, as it has also been observed in blunt force trauma on the compression side [24].

The general cortical traits plastic deformation, fracture surface scaling and marginal chipping occurred in 100% of all sample groups. It is conceivable that these traits are not influenced by the impact location or angle but are instead characteristic of long bone gunshot trauma in general. In the literature, plastic deformation is typically associated with low-velocity impacts. It has been widely reported that in blunt force trauma, bone behaves like a ductile material because it deforms before it breaks, while in gunshot trauma, bone behaves like a brittle material because it breaks immediately [33, 35, 36]. Our results do not support this general assumption. Indeed, other researcher have also emphasised that limited plastic deformation can occur in gunshot trauma too [35]. Hereby, it is, however, assumed that this phenomenon is associated to ballistic impacts at rather low velocities [14, 17]. Fracture surface scaling and marginal chipping have not been described in human gunshot fractures previous to Schwab et al.’s work [22]. With respect to the latter, a similar finding of missing bone chips at the fracture margin has been described in repeated blunt force trauma [25]. There, it has been explained by neighbouring fractures rubbing against each other in repeated impacts, which eventually causes a spalling at the bone edges.

### The anterior group

As the fracture pattern in perpendicular anterior shaft shots has been explained in the earlier study by Schwab et al. [22], we will focus here only on the differences compared to the other sample groups. In this regard, we observed that stepped breakout was significantly more common in the Anterior group than in the others. This potentially supports the previously proposed hypothesis that its formation might be influenced by the dense and edgy *linea aspera*, since it was more common in femurs than humeri [22]. Contradicting this hypothesis, the fact that the Anterior angled group showed an occurrence as low as the Lateral group may not support it. Another notable finding is that square exit hole was most consistently reproduced in the Anterior group. A potential link to the *linea aspera*, resulting in a more irregular exit defect, might be discussed for this trait, too. This assumption finds support by our observation that the Angled anterior group also predominantly exhibited square exit holes, whereas the Lateral and Posterior groups did not. Furthermore, we observed that entry-associated concentric fractures, wing flake defect and lateral notch were most consistently reproduced in the Anterior group, though, not statistically significant.

### The lateral group

The strong similarity between the Lateral and Anterior group may be explained by the similarity in the two shooting scenarios: a perpendicular, centred shot towards a rather regular and rounded surface. In the other groups, the impacts were either angled, tangential or towards the *linea aspera*. Nonetheless, the Lateral group tended to have lower occurrence values than the Anterior group. With respect to the quantitative characteristics, the Lateral group presented the highest values for all variables: vertical and horizontal entry and exit hole diameters as well as fracture extent. It is particularly interesting that significant differences were found in the vertical and horizontal entry and exit hole diameters between the Lateral and Anterior group, despite the similarity of both in the qualitative traits. The lateral shaft side is more curved than the anterior, but whether this geometry explains the discrepancies requires further research.

### The anterior angled group

At first sight, the Anterior angled group appeared remarkably similar to the Anterior group. A closer look, though, allowed for a distinction between the perpendicular and angled shots. Whilst in the Anterior group, the bullet entry and exit were on a similar level, in the Anterior angled group the exit was further distal than the entry. This can be understood by the trajectory of the bullet from top to bottom. In some samples, the bullet also exited slightly lateral to the entry, suggesting that the bullet deviated more than in perpendicular impacts. Another notable difference compared to the perpendicular shots was that in the Anterior angled group, tip fragmentation was exclusively limited to the distal part of the entry. Moreover, ring defect appeared considerably subtle proximal to the entry. Hence, the cortical damage on the side of the smaller impact angle seems to be lower than on the side of the greater impact angle. More difficult to explain is the reduced occurrence of exit-associated longitudinal concentric fractures, the absence of grey discolouration, and the shortest fracture extent in the group comparison. The influence of the impact angle on those characteristics may be further studied.

### The posterior group

The peculiarity of the Posterior group is that the bullet entered a particularly dense and edgy region of the shaft, the *linea aspera*. This might be an important factor for the conspicuous fracture characteristics in this group. On the one hand, the fracture pattern was characterised by a comparably low occurrence of entrance and exit traits such as internal bevelling, concentric fractures, V-shape, wing flake, wing flake defect, square hole and stepped breakout. On the other hand, grey discoloration was most pronounced in the Posterior group with a 100% occurrence. In this context, it is conceivable that the bullet casing is more likely to break upon impact with denser structures like the *linea aspera*. This may increase the likelihood that the inner lead core becomes exposed and the bone coloured.

### The grazing group

The major characteristic of the grazing fracture was that only one single defect hole was reproduced, a merged bullet entry and exit. In this context, the absence of distinct exit defects, as reported in the literature, warrants further discussion. Authors have assumed that in long bone gunshot trauma, the entry fractures travel fast enough to break the opposite shaft side before the bullet exits [16, 17]. We cannot exclude such a phenomenon, but it is conceivable that a grazing fracture might be misinterpreted as a bullet entry with missing exit. This particularly applies when the shooting circumstances are unknown. In line with other authors, we observed that one part of the grazing hole exhibits entry and the other part exit characteristics [37]. In comparison to the other groups, though, with generally lower occurrence values and some peculiarities. In craniums, grazing shots have been described to result in keyhole fractures, consisting of a rounded and triangular part [38]. The first is formed by the bullet entry, and the second by the exit. Similarly, we also found parts of a round entry hole at the impact side, whilst the exit was more irregular. Furthermore, the degree of cortical defects at the entry hole margin was rather low. Ring defect could typically be found, but was comparatively subtle. Tip fragmentation was significantly less common than in the other groups, and wing flake defect with wing flake never occurred. Whilst radiating fractures were always present, concentric fractures were not observed at the bullet entry. This might be associated to a reduced blast effect in comparison to shaft shots with a more centred impact [22, 39]. Internal bevelling and wing piece proved to be typical traits of the grazing fractures, less frequently also lateral notch and v-shape. These four traits, however, revealed to be potentially misleading in determining the bullet direction. In the Grazing group, their relation to the bullet’s entry and exit differed from that in the other groups. For instance, internal bevelling appeared at the entry hole, but the funnel-shaped cortical opening pointed towards the inner shaft instead of the bullet’s flight direction. This suggests that in grazing shots, internal bevelling does not indicate the bullet’s flight direction, as it usually does [40–43]. Regarding wing piece and v-shape, the manner they formed around the grazing hole created the false impression of a bullet entry. In concrete, wing piece appeared at the exit part of the grazing hole, and correspondingly also lateral notch. This indicates that wing piece and lateral notch cannot be classified as classic entry traits in grazing fractures. Similarly, also v-shape cannot be considered as an entry trait in grazing fractures. In contrast to the other sample groups, v-shape was formed by a radiating fracture of the entry and exit, each. Hence, it appeared sideways to the bullet trajectory, pointing with its tip towards the transition of bullet entry and exit (instead of the bullet entry). Complicating matters, we also observed tip fragmentation on the v-shape, reinforcing the false impression of an entry hole.

With respect to quantitative characteristics, the mean vertical entry hole diameter appeared smaller than the bullet diameter. This differed from the other groups. Nonetheless, since the tangential impact does not reproduce an entire entry hole, it is conceivable that the vertical diameter in grazing shots does not reflect the maximum potential diameter. Thus, even more caution is required when trying to deduce the bullet calibre from grazing shots.

The exit part of the grazing hole was more irregular than the entry part, but did not allow us to accurately classify the shape or measure the diameter. In accordance with descriptions on keyhole defects, the exit side revealed a superficial cortical damage [37, 38]. Yet, in contrast to Berryman and Gunther [37], we did not refer it as to external bevelling, because it did not present as a funnel-shaped opening that affected the entire cortical wall. Its appearance can potentially also be confused with wing flake defect. A closer look, however, reveals a rather rough and irregular defect surface, indicating that multiple tiny bone fragments broke out. In comparison, a true wing flake defect has a fairly smooth surface, matching to a flat trapezoidal piece of bone (wing flake) being pushed-off. Furthermore, the exit part of the grazing hole featured multiple radiating fractures that travelled in the direction of the bullet, which was also reported by Berryman and Gunther [37]. Although exit-associated concentric fractures occurred in the Grazing group, they did not multiply and thus, not result in stepped breakout, which is in contrast to the other groups.

### Limitations

To ensure accurate interpretation, several limitations must be addressed. While the use of real human bones provided a unique opportunity, the sample size was small, with most bones donated by men aged 44 to 74. Age- and sex-related bone properties may affect fracture patterns, limiting the results’ generalisability. Furthermore, previous research indicated slight differences in ballistic fracture patterns between femurs and humeri [22]. Since this study focused solely on femurs, caution is needed when applying the findings to other long bones. Another limitation concerns the use of Clear Ballistics Gel^®^. While it offers advantages in ease of handling and excellent transparency, allowing for precise shots, it is not equivalent to the standard 10% ballistic gelatine and does not meet FBI calibration criteria.

## Conclusions

The findings suggest that the previously characterised cortical traits in ballistic long bone trauma are also reproducible when the bullet’s impact angle and impact location on the shaft vary. By and large, the fractures appeared similar, except for the more complex grazing fracture. General cortical traits like plastic deformation, marginal chipping and fracture surface scaling suggest to be commonplace characteristics of ballistic long bone trauma, while the occurrence of most other traits may vary depending on the shooting scenario. The Anterior and Lateral group showed the most similar fracture pattern, likely due to the shared perpendicular, centered impact on a rounded surface, whereas the other shots were either angled, tangential or onto the *linea aspera*. Indeed, the results highlight the *linea aspera’s* potential influence on the fracture pattern. As demonstrated in the Posterior group, bullet entry through the *linea aspera* is possibly related to a higher occurrence of grey discolouration and a lower occurrence of internal bevelling, v-shape, wing flake, wing flake defect and concentric fractures. Conversely, bullet exit through the *linea aspera*, may rather lead to square exit hole and stepped breakout, as suggested by the anterior shots. Furthermore, the results from the Anterior angled group suggest that the fracture allows certain conclusions to be drawn about the impact angle. Corresponding to the bullet trajectory, the level of the exit hole may vary compared to the entry. In addition, a smaller impact angle appeared to cause less superficial bone damage around the entry hole, as indicated by a subtle ring defect and the absence of tip fragmentation. Lastly, the Grazing group produced the most dissimilar fracture. Even though most qualitative traits were found, lower occurrences and some peculiarities in their appearance were observed. Unlike the other groups, the grazing shots did not exhibit separate entry and exit holes. Instead, they showed a single grazing hole, with one part displaying entry characteristics and another part displaying exit characteristics. A careful assessment seems crucial to avoid misinterpreting a grazing fracture as a shaft fracture with missing bullet exit.

Taken together, these findings elucidate the similarity and variability of ballistic fracture patterns in human long bones and provide a basis for an improved interpretation. Determining the fracture characteristics cannot only help to establish the bullet’s trajectory but may also reveal conclusions on the bone’s position at the moment of impact. Such directional and positional information may assist in reconstructing events or even assessing statements provided to law enforcement by involved parties.

Nonetheless, despite the novel insights provided by this research, caution is needed due to influencing factors like bone properties, impact dynamics, and ballistic variables. Such factors may introduce variability, especially in complex fractures, and should be considered to avoid overinterpretation of the results. Further research is necessary to assess how potential influencing variables may alter the fracture pattern.

## Electronic supplementary material

Below is the link to the electronic supplementary material.

Supplementary Material 1

Supplementary Material 2
