# Supplementary material for: Fracture patterns in diaphyseal gunshot trauma: role of the bullet’s impact point and angle
Source: Int J Legal Med. 2025 Apr 7;139(5):2189–206. doi: 10.1007/s00414-025-03488-0 (PMC12354611; doi:10.1007/s00414-025-03488-0)
Supplement: Supplementary file 1 — Supplementary Material 1 [file 414_2025_3488_MOESM1_ESM.docx]

Fracture patterns in diaphyseal gunshot trauma: Role of the bullet’s impact point and angle

Nathalie Schwab^1,2,3^, Doreen Jost^2,4^, Xavier Jordana*^3,5^, Jordi Monreal^6^, Xavier Garrido^6^, Pedro Brillas^7^, Ignasi Galtés*^2,8^

**Affiliations:**

1. Institute of Legal Medicine St.Gallen, HOCH Health Ostschweiz, Cantonal Hospital, University teaching and research hospital, 9007 St.Gallen, Switzerland
2. Forensic Anthropology Unit, Forensic Pathology Service, Catalonian Institute of Legal Medicine and Forensic Science (IMLCFC), Ciutat de la Justícia, Gran Via de les Corts Catalanes, 111 Edifci G, 08075 Barcelona, Spain
3. Biological Anthropology Unit, Department of Animal Biology, Plant Biology and Ecology, Faculty of Biosciences, Universitat Autònoma de Barcelona, Cerdanyola del Vallès, 08193 Barcelona, Catalonia, Spain
4. Institute for Interdisciplinary Studies, Faculty of Science, University of Amsterdam, Science Park 904, 1098 XH Amsterdam, The Netherlands
5. Tissue Repair and Regeneration Laboratory (TR2Lab), Institut de Recerca i Innovació en Ciències de la Vida i de la Salut a la Catalunya Central (IrisCC), Ctra. de Roda, 08500 Vic, Barcelona, Spain
6. Mossos d’Esquadra, Unitat Central de Balística i Traces Instrumentals, Av. de la Pau, 12, 08206 Sabadell, Barcelona, Spain
7. Donor Center Barcelona Tissue Bank (BTB), Hospital Clínic de Barcelona, C/Villarroel 170, Escala 12 Planta 4, 08036 Barcelona, Spain
8. Research Group of Biological Anthropology (GREAB), Biological Anthropology Unit, BABVE Department, Universitat Autònoma de Barcelona (UAB), Cerdanyola del Vallès, 08193 Bellaterra, Catalonia, Spain

***Corresponding authors:**

**Ignasi Galtés**

E-mail: [ignasigaltes@gmail.com](mailto:ignasigaltes@gmail.com)

**Xavier Jordana**

E-mail: [xavier.jordana@uab.cat](mailto:xavier.jordana@uab.cat)

**Online Resources 1a-c**

**1a Definition of the cortical entrance traits**

| ***Entrance trait*** | ***Definition*** |
| --- | --- |
| Round entry hole | The projectile’s initial entry defect appearing visually round. |
| Radiating fracture | Longitudinal, transversal or oblique fractures that radiate away from the entry hole. |
| Concentric fracture | Curvilinear fractures facing with their concave aspect towards the entry hole. They can be divided into transversal or longitudinal ones. The first occurs proximal/distal to the entry hole, the second on the sides of the entry hole. |
| V-shape | Describes the shape of the shaft proximal/distal to the bullet entry/trajectory. |
| Ring defect | A circular or partial circular loss of superficial cortical bone on the entry hole’s margin. Its presentation reminds of an abrasion ring. It can be interrupted by other entrance traits such as tip fragmentation. |
| Tip fragmentation | A superficial, external loss of cortical chip(s) at the tip of the bone around the entry hole. It only appears proximal/distal to the bullet entry. Typically, chiselled edges indicate a sharply defined transition to the intact cortical bone. In case oblique fractures separate the bone, tip fragmentation on bone parts next to each other creates the image of a stepped circular margin around the bullet entry. |
| Wing flake | A loose trapezoidal flake of cortical bone resembling spread wings. The aspect facing the entry hole features part of the ring defect. In contrast to tip fragmentation, it comes from the lateral aspect(s) of the entry hole. It may be broken and thus incomplete. |
| Wing flake defect | The superficial cortical defect on the lateral aspect(s) of the entry hole that is produced when the wing flake breaks out. |
| Wing piece | A characteristic bone fragment with the shape of spread wings, similar to the wing flake, but usually much bigger and most importantly, affecting the entire cortical wall. Only the extremes can run out superficially. The aspect facing the entry hole may contain part of the ring defect or a wing flake defect. It can be entire or broken into two or more fragments. Less frequently, it may be still attached to the shaft on one side. |
| Lateral notch | As the wing piece’s extremes run out superficially, they leave behind a corresponding cortical defect on the lateral shaft aspect. On this fracture surface, there is a prominent notch visible. |
| Internal bevelling | A funnel-shaped opening in the cortical wall with the larger part on the inside. |

**1b Definition of the cortical exit traits**

| ***Exit trait*** | ***Definition*** |
| --- | --- |
| Square exit hole | The projectile’s exit defect appears visually edgy or square. |
| Radiating fracture | Transversal, longitudinal or oblique fractures that radiate away from the exit hole. |
| Concentric fracture | Curvilinear fractures facing with their concave aspect towards the exit hole. They can be divided into transversal or longitudinal ones. The first occurs proximal/distal of the exit hole, the second on the sides of the exit hole. |
| Layered breakage | Is a layered pattern visible on the fracture surface. |
| External bevelling | A funnel-shaped opening in the cortical wall with the larger part on the inside. |
| Stepped breakout | A sequence of multiple transversal concentric fractures on the posterior shaft aspect resulting in a stepped breakout of fragments with similar shapes. |

**1c Definition of the general cortical traits**

| ***General trait*** | ***Definition*** |
| --- | --- |
| Plastic deformation | Is the persistent deformation of bone. |
| Marginal chipping | Tiny, superficial cortical defects on the fracture margin. |
| Fracture surface scaling | Tiny cortical scaling on the fracture surface. |
| Grey discolouration | Traces of grey colour on the bone in the area of the bullet trajectory. |
