# Supplementary material for: Fracture patterns in diaphyseal gunshot trauma: role of the bullet’s impact point and angle
Source: Int J Legal Med. 2025 Apr 7;139(5):2189–206. doi: 10.1007/s00414-025-03488-0 (PMC12354611; doi:10.1007/s00414-025-03488-0)
Supplement: Supplementary file 2 — Supplementary Material 2 [file 414_2025_3488_MOESM2_ESM.docx]

**Fracture patterns in diaphyseal gunshot trauma: Role of the bullet’s impact point and angle**

Nathalie Schwab^1,2,3^, Doreen Jost^2,4^, Xavier Jordana*^3,5^, Jordi Monreal^6^, Xavier Garrido^6^, Pedro Brillas^7^, Ignasi Galtés*^2,8^

**Affiliations:**

1. Institute of Legal Medicine St.Gallen, HOCH Health Ostschweiz, Cantonal Hospital, University teaching and research hospital, 9007 St.Gallen, Switzerland
2. Forensic Anthropology Unit, Forensic Pathology Service, Catalonian Institute of Legal Medicine and Forensic Science (IMLCFC), Ciutat de la Justícia, Gran Via de les Corts Catalanes, 111 Edifci G, 08075 Barcelona, Spain
3. Biological Anthropology Unit, Department of Animal Biology, Plant Biology and Ecology, Faculty of Biosciences, Universitat Autònoma de Barcelona, Cerdanyola del Vallès, 08193 Barcelona, Catalonia, Spain
4. Institute for Interdisciplinary Studies, Faculty of Science, University of Amsterdam, Science Park 904, 1098 XH Amsterdam, The Netherlands
5. Tissue Repair and Regeneration Laboratory (TR2Lab), Institut de Recerca i Innovació en Ciències de la Vida i de la Salut a la Catalunya Central (IrisCC), Ctra. de Roda, 08500 Vic, Barcelona, Spain
6. Mossos d’Esquadra, Unitat Central de Balística i Traces Instrumentals, Av. de la Pau, 12, 08206 Sabadell, Barcelona, Spain
7. Donor Center Barcelona Tissue Bank (BTB), Hospital Clínic de Barcelona, C/Villarroel 170, Escala 12 Planta 4, 08036 Barcelona, Spain
8. Research Group of Biological Anthropology (GREAB), Biological Anthropology Unit, BABVE Department, Universitat Autònoma de Barcelona (UAB), Cerdanyola del Vallès, 08193 Bellaterra, Catalonia, Spain

***Corresponding authors:**

**Ignasi Galtés**

Forensic Anthropology Unit, Forensic Pathology Service, Catalonian Institute of Legal Medicine and Forensic Science (IMLCFC)

Ciutat de la Justícia. Gran Via de les Corts Catalanes, 111 Edifici G, 08075 Barcelona, Spain

Tel. 00-34-93 554 82 78

E-mail: [ignasigaltes@gmail.com](mailto:ignasigaltes@gmail.com)

**Xavier Jordana**

Biological Anthropology Unit, Department of Animal Biology, Plant Biology and Ecology

Faculty of Biosciences, Universitat Autònoma de Barcelona (UAB)

Cerdanyola del Vallès, 08193 Barcelona, Catalonia, Spain

E-mail: [xavier.jordana@uab.cat](mailto:xavier.jordana@uab.cat)

**16-digit ORCID:**

Nathalie Schwab: 0000-0003-4379-3509

Ignasi Galtés: 0000-0002-5758-2668

Xavier Jordana: 0000-0002-6016-6630

**Abstract:**

Skeletal trauma assessment is an important task of forensic anthropologists and pathologists. This applies in particular to badly preserved bodies where the soft tissue cannot provide forensic evidence. Yet, the interpretation of ballistic long bone trauma can be difficult due to little conclusive data. Thus, this study explored the variability of diaphyseal fracture patterns dependent on the bullet’s angle and point of impact. 20 femurs from body donors were embedded in Clear Ballistics Gel® and divided into 4 experimental groups: 70° angled shot on the centre of the anterior shaft aspect; perpendicular shot on the centre of the lateral shaft aspect; perpendicular shot on the centre of the posterior shaft aspect; grazing shot from posterior on the margin of the medial shaft aspect. In each case, a 9-mm Luger full metal jacket projectile was shot at a distance of 2 m and an impact speed of 360 m/s. All fractures were examined macroscopically. For the trauma comparison, a fifth group (perpendicular shot on the centre of the anterior shaft aspect), previously analysed in an earlier study, was included. Although the groups revealed similar fracture characteristics, the results suggest the bullet’s impact angle and location influence the fracture pattern. The most dissimilar fracture pattern was reproduced in the grazing shots, where only one defect hole was seen, instead of an entry and exit hole like in the other groups. The findings highlight the variability of ballistic fracture patterns in long bones and may serve as guidelines during the skeletal trauma assessment.

**Keywords:**

Forensic anthropology, Human bones, Long bones, Femur, Gunshot trauma, Ballistic fracture pattern

**Statements and declarations**

**Author contributions:**

Nathalie Schwab and Doreen Jost contributed equally to the work and are shared first authors. Doreen Jost is a junior scientist.

Conceptualisation: [Nathalie Schwab, Doreen Jost, Ignasi Galtés]; Methodology: [Doreen Jost, Nathalie Schwab, Jordi Monreal, Xavier Garrido, Pedro Brillas]; Formal analysis and investigation: [Doreen Jost, Nathalie Schwab, Ignasi Galtés, Xavier Jordana]; Writing - original draft preparation: [Nathalie Schwab, Doreen Jost]; Writing - review and editing: [Ignasi Galtés, Xavier Jordana, Nathalie Schwab]; Visualization: [Doreen Jost, Nathalie Schwab, Ignasi Galtés, Xavier Jordana]; Supervision: [Ignasi Galtés, Xavier Jordana, Nathalie Schwab].

**Competing Interests:**

The authors declare that they have no competing interests.

**Conflict of interest:**

The authors have no conflicts of interest to declare that are relevant to the content of this work.

**Funding Declaration:**

Proyectos de Generación de Conocimiento, Agencia Estatal de Investigación (PID2021-124112NB-100).

Research Fund for Excellent Junior Researchers of the University of Basel, Switzerland.

**Compliance with ethical standards:**

This research followed the ethical precepts of the Declaration of Helsinki (Fortaleza, Brazil, Oct 2013). It was approved by the local ethics committee (Bellvitge University Hospital, L’Hospitalet de Llobregat, Barcelona, Spain; Ref. PR416/20). The human samples were processed according to the guidance for clinical use (EEC regulations 2004/23/CE and 2006/17/CE) and to the legal requirements of Spain (Law 14/2007, RD 1716/2011 and RD 9/2014). All human bones were donated anonymously and obtained under informed consent. All samples are stored in the private collection at the Institut de Medicina Legal i Ciències Forenses de Catalunya (IMLCFC) in Barcelona, Spain (Registro Nacional de Biobancos. Ref. C.0004241).

**Acknowledgments:**

The authors are grateful to the Mossos d’Esquadra for providing the facilities, the material and the personal staff in order to realize the gunshot experiments.
